# Photophore‐Anchored Molecular Switch for High‐Performance Nonvolatile Organic Memory Transistor

**DOI:** 10.1002/advs.202401482

**Published:** 2024-03-30

**Authors:** Syed Zahid Hassan, Jieun Kwon, Juhyeok Lee, Hye Ryun Sim, Sanghyeok An, Sangjun Lee, Dae Sung Chung

**Affiliations:** ^1^ Department of Chemical Engineering Pohang University of Science & Technology (POSTECH) Pohang 37673 Republic of Korea

**Keywords:** deep trap states, field‐effect transistors, molecular‐switch memory, photocrosslinking, polymeric semiconductor

## Abstract

Over the past decade, molecular‐switch‐embedded memory devices, particularly field‐effect transistors (FETs), have gained significant interest. Molecular switches are integrated to regulate the resistance or current levels in FETs. Despite substantial efforts, realizing large memory window with a long retention time, a critical factor in memory device functionality, remains a challenge. This is due to the inability of an isomeric state of a molecular switch to serve as a stable deep trap state within the semiconductor layer. Herein, the study addresses this limitation by introducing chemical bonding between molecular switch and conjugated polymeric semiconductor, facilitating closed isomer of diarylethene (DAE) to operate as a morphologically stable deep trap state. Azide‐ and diazirine‐anchored DAEs are synthesized, which form chemical bonds to the polymer through photocrosslinking, thereby implementing permanent and controllable trapping states nearby conjugated backbone of polymer semiconductor. Consequently, when diazirine‐anchored DAE is blended with F8T2 and subjected to photocrosslinking, the resulting organic FETs exhibit remarkable memory performance, including a memory window of 22 V with a retention time over 10^6^ s, a high photoprogrammable on/off ratio over 10^3^, and a high operational stability over 100 photocycles. Further, photophore‐anchored DAEs can achieve precise patterning, which enables meticulous control over the semiconductor layer structure.

## Graphical abstract

Recently there has been tremendous interest in molecular‐switch‐embedded memory devices. However, achieving a substantial memory window and prolonged retention time remains a challenge. The diazirine‐anchored DAE linker (DL2) offers promise with a milder photocrosslinking process, leading to improved performance. When combined with F8T2, it results in FETs with a 22 V memory window, over 10^6^ s of retention time, a significant ON/OFF ratio, and robust stability.

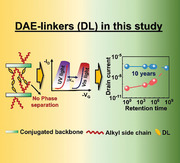

## 1 Introduction

Organic electronics, characterized by versatile performance, find applications across diverse electronic realms. Recent advancements in organic semiconductors (OSC) and innovative organic field‐effect transistor designs hint at their potential in logic applications,^[^ ^1^ ^]^ magnetosensing,^[^ ^2^ ^]^ bioelectronics,^[^ ^3^, ^4^ ^]^ and thermoelectrics.^[^ ^5^, ^6^ ^]^ However, the forefront of electronic research is currently dominated by memory and neuromorphic computing in the organic electronics domain.^[^ ^7^, ^8^, ^9^, ^10^, ^11^, ^12^, ^13^ ^]^ Organic field‐effect transistor (OFET) memory, among various configurations, emerges as a promising technology, offering lightweight, cost‐effective, and flexible charge storage solutions. In recent decades, significant advancements have been made in improving the performance of organic field‐effect transistors (OFETs), particularly in terms of charge carrier mobility and operational stability.^[^ ^14^, ^15^, ^16^, ^17^, ^18^, ^19^ ^]^ These advancements have spurred research into diversifying and enhancing the functionality of OFETs.^[^ ^20^, ^21^, ^22^ ^]^ One widely accepted approach for achieving multifunctionality in OFETs involves incorporating molecular switches capable of inducing transitions between two or more metastable isomers through light stimuli.^[^ ^23^, ^24^ ^]^ Previous studies have shown that integrating a molecular switch within an OSC facilitates effective control of trap levels, thereby resulting in photoprogrammable OFETs.^[^ ^25^, ^26^, ^27^, ^28^, ^29^ ^]^ Among the photochromic molecular switches commonly used in combination with OSCs, diarylethenes (DAEs) have emerged as a prominent choice. These switches feature functionalized aryl groups and ethylene bridges with diverse functional groups. DAE outperforms azobenzene and spiropyran in transistor‐based memory devices due to its unique electronic properties. Despite minimal geometric changes during photoisomerization, the aryl group of DAE enables efficient photomodulation of energy levels, making it an effective charge trapping center. The thermal stability in both open and closed states of DAE ensures prolonged memory retention, boosting reliability and durability in information storage.^[^ ^23^ ^]^ The open form of DAE (DAE_o) exhibits limited π‐conjugation within the aryl ring, whereas the closed form (DAE_c) demonstrates delocalized π‐conjugation throughout the molecule.^[^ ^30^ ^]^ Extended conjugation in DAE_c results in a reduced HOMO–LUMO gap, enabling it to function as trap states in OSCs. Both forms of DAEs exhibit remarkable bistable characteristics; thus, they are highly suitable for inducing trap levels in high‐performance photoprogrammable OFETs.^[^ ^31^ ^]^

The most widely adopted approach for fabricating OFETs with embedded DAE molecules is to blend DAEs with OSCs. Samori et al. successfully embedded DAEs into P3HT, resulting in 256 controllable photocurrent levels determined by light intensity.^[^ ^32^ ^]^ Other studies have shown that DAEs with larger steric volumes at the end of the aryl group exhibit better photocycloreversion or closed‐to‐open isomerization. This is because of the larger free volume available for isomerization.^[^ ^26^ ^]^ Additionally, it has been demonstrated that DAEs are more compatible with low‐crystalline OSC owing to the locking effect of highly crystalline semiconductors, such as poly[2,5‐(2‐octyldodecyl)‐3,6‐diketopyrrolopyrrole‐alt‐5,5‐(2,5‐di(thien‐2‐yl)thieno[3,2‐b]thiophene)] (DPP‐TT).^[^ ^33^ ^]^ These studies on DAE‐embedded OFETs have demonstrated high photoprogrammable I_DS_ ON/OFF ratios of over 10^3^ (the photoprogrammable I_DS_ ON/OFF ratio is defined as |I_DS_ (DAE_o)| / |I_DS_ (DAE_c)|) for blend systems.^[^ ^26^ ^]^ Another method for fabricating photoprogrammable OFETs with molecular switch involves the covalent incorporation of molecular switches into polymer semiconductors. Zhang et al. demonstrated the feasibility of this approach by introducing an azobenzene derivative into the side chains of a DPP‐based donor–acceptor copolymer.^[^ ^34^, ^35^, ^36^ ^]^ This approach resulted in a photoprogrammable polymer semiconductor. The photoprogrammable I_DS_ ON/OFF ratios achieved through this method were less than 10, which is considerably lower than those of the polymer:DAE mixture cases (>10^3^). However, the method is still promising for the development of photoprogrammable OFET devices because it can impart unique switching characteristics to the OSC backbone. The polymer:DAE mixture approach or bulk heterojunction (BHJ) approach has proven to be the most successful in terms of photoprogrammable I_DS_ ON/OFF ratios.^[^ ^37^ ^]^ However, all previous studies could not successfully demonstrate a photoprogrammable threshold voltage shift (ΔV_th_) or memory window in OFET memory, which is a pivotal factor in memory device functionality. Given that the ΔV_th_ is determined by deep trap states,^[^ ^38^, ^39^ ^]^ we can speculate that simply mixing a molecular switch with a polymer semiconductor is unlikely to induce stable deep trap state and more strategical approach is required. Actually, the mixing approach has limitations of phase stability due to the inherent susceptibility of the blends to phase separation.^[^ ^40^ ^]^ To address the phase stability issue in OFETs and other organic devices, a crosslinking strategy using azide,^[^ ^41^ ^]^ diazirine,^[^ ^42^ ^]^ benzophenone,^[^ ^43^ ^]^ diazo,^[^ ^44^ ^]^ and free‐radical crosslinkers,^[^ ^45^ ^]^ has been introduced. However, these efficient photocrosslinking methods cannot be utilized in DAE/OSC blend systems because of their inability to facilitate crosslinking between the small‐molecule DAE and polymeric OSC.

In this study, we designed and synthesized two novel photophore‐anchored molecular switches capable of functioning as both molecular switches and photocrosslinkers. Two different photophores of azide and diazirine were introduced as photocrosslinkable functional groups of DAE, resulting in 1,2‐bis‐(4‐methyl‐2‐propyl‐5‐(methylphenyl‐4‐azido‐2,3,5,6‐tetrafluorobenzoate)‐3‐thienyl)cyclopentene and 1,2‐bis‐(4‐methyl‐2‐propyl‐5‐(methylphenyl‐4‐[3‐(trifluoromethyl)‐3H‐diazirin‐3‐yl]benzoate)‐3‐thienyl)cyclopentene, named as DL1 and DL2, respectively. The primary goal was to induce DAE_c to function as morphologically stable deep trap states by introducing chemical bonding between DAE and the polymer semiconductor via photocrosslinking. This chemical bonding can bind the conjugated moiety of DAE and the conjugated backbone of a polymeric semiconductor against binodal decomposition. The fabricated memory OFETs consisting of poly(9,9‐dioctylfluorene‐alt‐bithiophene) (F8T2) as a semiconductor and DL1 or DL2 as a photophore‐anchored molecular switch resulted in a large memory window with sufficiently long memory‐retention time, and high photoprogrammable I_DS_ ON/OFF ratios. Additionally, the photocrosslinker demonstrated enhanced operational stability and fine photopatternability. A comprehensive range of techniques, including UV–vis spectrophotometry, fourier transform infrared (FTIR) spectroscopy, grazing incidence X‐ray diffraction (GIXD) studies, and optical microscopic imaging, were used to investigate the photochemical properties, crosslinking behavior, film robustness, structural analysis, and photopatternability of the DAE/OSC blend.

## 2 Results and Discussion

### 2.1 Synthesis and Characterization

**Figure 1:**
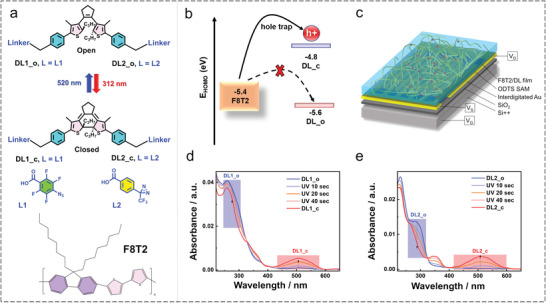
a) Chemical structures of the synthesized DLs, DL1, and DL2, in both their open‐ and closed forms. The arrows indicate the reversible photoswitching of DLs, with 312 nm and 520 nm wavelength light sources utilized for photocyclization and photocycloreversion, respectively. Additionally, the chemical structure of the commercially available polymer semiconductor, F8T2, is depicted, b) schematic illustration for the hole‐trapping mechanism between HOMO levels of F8T2 and DLs in their open and closed forms. This mechanism demonstrates the trapping of a hole after exposure to UV light and its detrapping upon irradiation with a visible light source, c) device geometry of photoprogrammable OFETs where F8T2 and DL molecules are represented by the yellow chain and the red sphere, respectively, in the F8T2 polymer matrix. UV–vis absorption spectra of d) F8T2/DL1 and e) F8T2/DL2 in solution states. The blue and red colors indicate the UV–vis absorption spectra before and after exposure to UV light (312 nm, 0.45 mW cm^−2^), respectively.

Two DLs were synthesized using azide (DL1) and diazirine (DL2) photophores. The structures of DL1 and DL2 are depicted in **Figure** **1**a, and their synthesis procedures are summarized in Scheme S1 (Supporting Information). The aim of designing and synthesizing these DLs was to utilize the HOMO of the closed and open isomers as trapping and detrapping sites, respectively, in conjunction with polymeric semiconductors (Figure 1b,c). To achieve this, we modified a previously reported molecule with an identical molecular backbone, which possessed exceptionally high fatigue resistance.^[^ ^28^, ^37^ ^]^ The solubility of the DLs was favorable in chloroform, chlorobenzene, dichlorobenzene, dichloromethane, ethyl acetate, acetone, and toluene; however, moderate solubility was observed in hexane, acetonitrile, and methanol.

The synthesis of DLs involves DCC coupling between an azide/diazirine, containing a –COOH moiety, and a DAE compound, containing two –OH groups. The synthesis of DAE with dual –OH groups is a multistep process involving chlorination, acylation, McMurry reaction, boronate formation, Suzuki coupling, and reduction. Upon exposure to UV light, both DL1 and DL2 exhibited a noticeable change in color, indicating the persistence of intact DAE moieties, even in the presence of azide and diazirine functionalities. The UV–vis absorption spectra of the DLs in the solution and film states were analyzed, and the results are summarized in Figure 1d,e and Figure S1a,b (Supporting Information). In the solution state, the UV–vis absorption spectra of DL1 and DL2 exhibited an absorption band between 220 and 350 nm, attributed to the presence of the open form of DAE (DAE_o).

To investigate the formation of the closed form of both DLs, a solution of the DLs (presumably referring to the DAE compounds) was irradiated with UV light at a wavelength of 312 nm. This irradiation resulted in the appearance of a new absorption peak in the range of 400–600 nm, indicating the successful synthesis of DAE compounds anchored with azide and diazirine groups.

The DLs were extensively characterized using ^1^H NMR, ^13^C NMR, and ^19^F NMR. F8T2 was selected as the OSC due to its favorable amorphous nanostructure, which is crucial for the successful photoisomerization of DAE within polymeric OSC, as verified by several previous studies.^13^, ^33^ The sufficiently large conformational flexibility of F8T2 provides unrestricted isomerization of DAE during the ring‐opening process, which is a key factor in enabling multiple photocycles within polymeric OSCs. In contrast, polymers such as DPP‐TT and IIDDT‐C3, which possess a high degree of crystallinity, are likely to limit the ring‐opening activity of DAEs.^[^ ^26^ ^]^

### 2.2 Photocrosslinking Reaction

**Figure 2:**
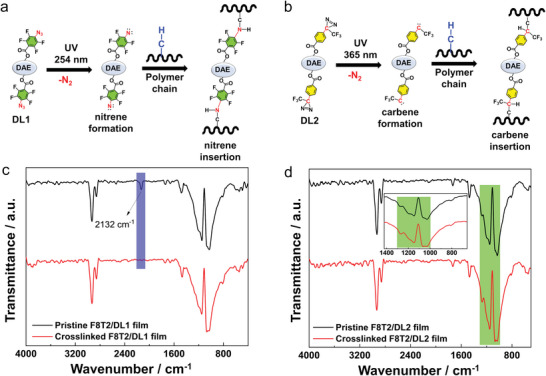
a) Mechanism for photocrosslinking using azide in DL1. Under UV irradiation, DL1 generates a nitrene intermediate that covalently crosslinks to the polymer target, b) mechanism for photocrosslinking using diazirine in DL2. When exposed to UV light, the diazirine generates a carbene intermediate, leading to the covalent crosslinking with the polymer target, c) FTIR spectra of pristine F8T2/DL1 film and crosslinked F8T2/DL1 film. The blue shaded box highlights the disappearance of the azide peaks after UV exposure, indicating a successful crosslinking process, and d) FTIR spectra of pristine F8T2/DL2 film and crosslinked F8T2/DL2 film. The green shaded box indicates the reduction of the −CF_3_ group peaks after UV exposure, signifying a successful crosslinking process. The inset displays an enlarged view of the peak occurring between 700 and 1400 cm^−1^.

**Figure** **2**a,b provide a schematic illustration of the photocrosslinking process of polymeric OSC using DLs. Upon exposure to UV light, the azide and diazirine groups within DL1 and DL2, respectively, underwent photolysis. This irradiation generated reactive singlet nitrene and carbene species, along with the release of N_2_ molecules. The crucial reaction between OSC and DLs involves the insertion of singlet nitrene and carbene into the C–H bonds of neighboring alkyl chains within the polymers, leading to a crosslinked network structure. Notably, DL1 requires deep UV light (254 nm) for successful photocrosslinking (Figure 2a), while DL2 can be efficiently photocrosslinked with milder UV (365 nm) light (Figure 2b). The FTIR spectra of the F8T2/DLs blend films, shown in Figure 2c,d, exhibit changes before and after UV exposure. Upon irradiation with 254 nm UV light, the disappearance of the distinctive vibrational peak corresponding to the azide group in DL1 at 2128 cm^−1^ confirms the successful occurrence of crosslinking (Figure 2c). To confirm the effective crosslinking of DL2, which includes a diazirine group, the FTIR spectrum depicted in Figure 2d clearly demonstrates a significant alteration in the vibrational peak associated with the CF_3_ group, falling within the range of 1030–1230 cm^−1^. This reduction in the intensity of the CF_3_ peak indicates that the generation of carbene species arising from the direct interaction with the diazirine groups significantly affects the vibration intensity of the CF_3_ moiety.^[^ ^47^ ^]^

While the FTIR spectra of the F8T2/DLs blend films do not clearly indicate the destruction of the DAE conjugation structure by the highly photoreactive azide and diazirine, the possibility of such damage should be considered. Observations from the UV–vis absorption spectra of pure DL films exposed to UV light and FTIR analyses (Figure S1, Supporting Information) revealed that the photoreactive azide and diazirine groups have the potential to cause degradation in the DAE conjugation structure. This is indicated by the disappearance of the photophore vibration peaks and a reduction in the intensity of the C–H vibrations of the DAE backbone. These findings suggest that the photoswitching property of the DAE linker was lost after the harsh UV exposure. However, the potential damage to the DAE conjugation structure by the photophores may not prevail in the F8T2/DL blend films due to the more efficient photocrosslinking reaction between the photophores and the alkyl chains of F8T2. The blended films created a different environment in which the photophores were more effectively engaged in the crosslinking process, leading to a higher degree of polymer network formation. This efficient crosslinking reaction is expected to protect the DAE conjugation structure from significant degradation.

Additionally, the FTIR spectra of the pristine F8T2 samples before and after UV irradiation of wavelengths 254 nm and 365 nm were compared (Figure S2a, Supporting Information). The intensity of the in‐plane C‐H bending vibrations within the aromatic ring (ranging from 1000 to 1200 cm^−1^) in the pristine F8T2 film decreased more noticeably when exposed to 254 nm UV light compared to its exposure to 365 nm UV light. Furthermore, UV–vis spectra exhibited a similar trend, with the pristine F8T2 film sample displaying absorption maxima at 456 nm and a shoulder peak at 484 nm, closely resembling the sample exposed to 365 nm UV light (Figure S2b, Supporting Information). In contrast, the sample exposed to 254 nm light showed a slightly reduced absorption peak for F8T2. As a result, employing DL2 for photocrosslinking in the F8T2/DAE system offers advantages over DL1.

This is because 365 nm UV light causes less damage to both the DAE and F8T2 conjugated backbones, thereby facilitating higher photoprogrammable performance after photocrosslinking. Note that the intensity of the UV light used for irradiation was same to that used in the crosslinking reaction. However, in this case, the samples were exposed to the UV light for a duration of 1 h. Moreover, the existence of a bulky CF_3_ group bonded to the carbon atom, which subsequently generates carbene in DL2 acts as a hindrance that prevents unintended carbene insertion reactions into the conjugated backbone of F8T2/DL2 blend system. This susceptibility could otherwise be readily taken advantage of by nitrene, as nitrene lacks a corresponding bulky group on its nitrogen atom, the source of nitrene formation.

### 2.3 Photoisomerization

The open form of DAE, present in both DLs, exhibited λ_max_ near 300 nm, which overlapped with the absorption band of azide in the case of DL1 and appeared as a shoulder band. Similarly, DL2 also exhibited a shoulder band, suggesting that the λ_max_ of azide and diazirine had a higher extinction coefficient, causing the λ_max_ of DAE to appear as a shoulder band. For achieving complete photoconversion of DL1 to its closed form, known as the photostationary state (PSS), the DL1 solution was irradiated with 312 nm UV light (Figure 1d). To investigate the reversibility of photoisomerization in DL1, light of wavelength 520 nm was irradiated, resulting in the successful disappearance of the absorption peak corresponding to the closed form of DAE at 509 nm. This observation confirms that in the solution state, even in the presence of azide, DAE is capable of undergoing reversible photoisomerization.

Similarly, to achieve complete conversion of the open form of DL2, 312 nm UV light was irradiated, resulting in the emergence of a peak at 512 nm, which corresponded to the closed form of DL2 (Figure 1e). Further, to assess the reversibility of photoisomerization in DL2, 520 nm light was irradiated, leading to the successful disappearance of the absorption band associated with the closed form at 512 nm. This finding confirms that even in the presence of diazirine, DAE in solution is capable of undergoing successful reversible photoisomerization. Notably, the molecular extinction coefficient of DL2 in its closed form is higher than that of DL1. This observation suggests that the formation of the carbene in DL2 is less prone to disturbing the molecular backbone of DAE than the nitrene generated in DL1. The nitrene leads to molecular backbone degradation, resulting in a reduced molar extinction coefficient for the closed form of DAE, even when in a solution state.

Observing photoisomerization in the neat film state poses challenges for both DL1 and DL2 due to the significant destruction of the molecular backbone of the DAEs caused by the photogenerated nitrene and carbene. However, successful photoisomerization reactions were observed when DLs were blended with F8T2. To investigate the photochemical processes of the DAE linkers within the F8T2 polymer matrix, samples were prepared by spin‐coating a binary solution of F8T2 and DLs at a concentration of 5 mg mL^−1^. The resulting samples were thermally annealed at 120 °C to enhance microstructural ordering. The blending ratio of DL and F8T2 was fixed at 20 wt.% DL based on previous studies.^[^ ^26^ ^]^

**Figure 3:**
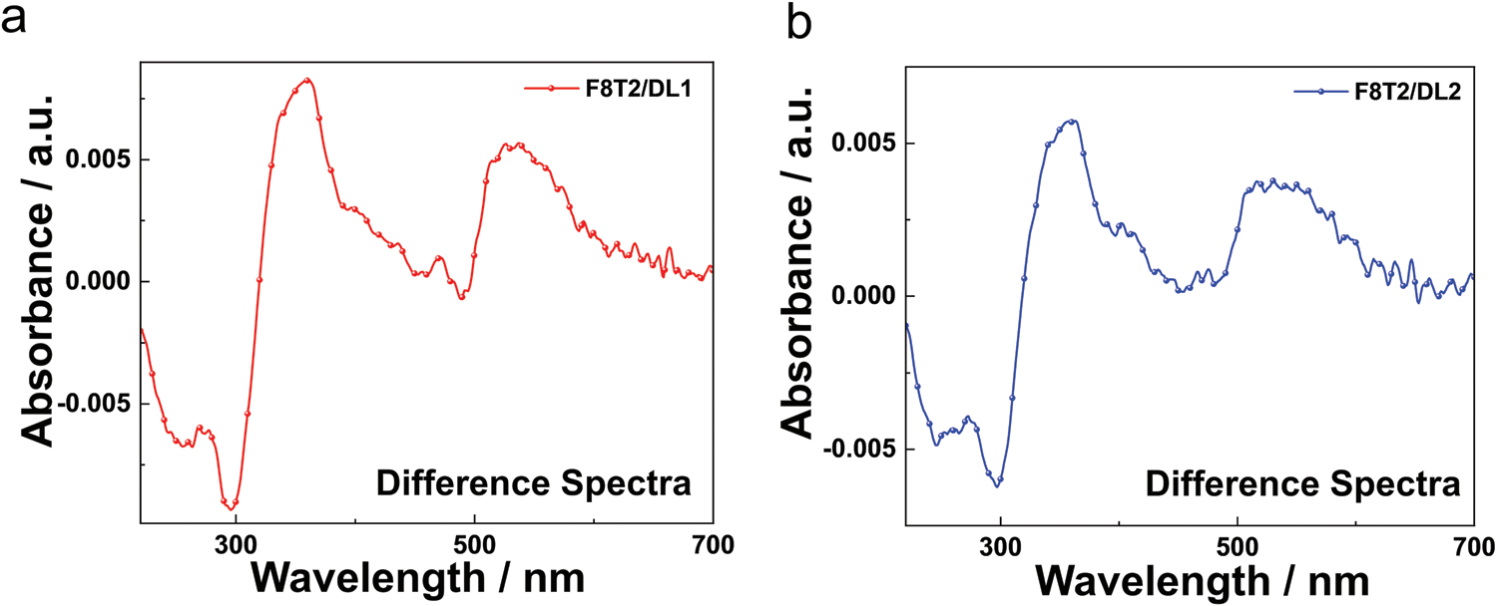
The differential changes in UV–vis absorption spectra for F8T2/DL blend films before and after UV light irradiation (312 nm): a) F8T2/DL1 film b) F8T2/DL2 film.

**Figure** **3**a,b depict the differences in UV–vis spectra of thin films comprising photocrosslinked DLs mixed with F8T2 obtained before and after the UV light irradiation. These films were irradiated with 312 nm for both DL1 and DL2 to induce photoisomerization. Upon exposure to irradiation, a noticeable enhancement was observed in the absorption band spanning at 400–600 nm, accompanied by a corresponding decrease in the absorption band at 220–370 nm. This outcome serves as a clear indication that the process of photoisomerization was successfully initiated. Notably, DL1 or DL2 did not induce any shift in the absorption maxima of F8T2. This outcome underscores that both DLs do not hinder the crucial π–π interaction of the F8T2 polymer. This interaction is pivotal for maintaining charge carrier mobility at levels akin to the pristine F8T2 polymer.

### 2.4 Microstructural Ordering

To analyze the film structures, we conducted 2D GIXD assessments on photocrosslinked F8T2/DL1 and F8T2/DL2 films, both prepared with a 20 wt.% loading ratio of DL. Interestingly, the positions of the diffraction peaks remained consistent with those observed in the pristine F8T2 film, except that there was a conspicuous increase in peak intensity by adding DLs (Figure S3, Supporting Information). It is worth mentioning that this 20 wt.% DL loading condition reproduces the optimal setup for F8T2 photopatterning with minimal loss of electrical characteristics.

### 2.5 Photoprogrammable Memory OFET

**Figure 4:**
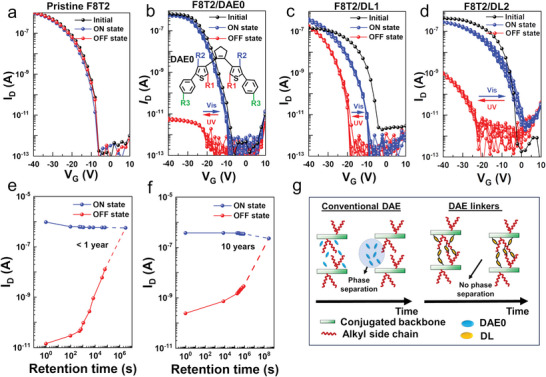
Photoprogrammed switching performance of F8T2/DL OFET devices: transfer curves of the OFET based on the a) pristine F8T2, b) F8T2/DAE0, inset shows the chemical structure of DAE0 which possesses R1 = C_3_H_7_, R2 = CH_3_, R3 = C_6_H_13_, c) F8T2/DL1, and d) F8T2/DL2 active layers for alternate steps of UV (312 nm) and visible (520 nm) light irradiation up to several steps. Retention characteristics of the e) F8T2/DAE0 OFET devices, f) F8T2/DL2 OFET devices, measured at a V_G_ of −40 V and V_D_ of −20 V. Dash line represents the extrapolation up to which each state can be distinguished, g) the schematic representation demonstrates the phase stability of a conventional DAE with a blended polymer, which exhibits instability over extended periods. In contrast, the DAE‐linkers synthesized in this study exhibit remarkable stability over prolonged durations, resulting in significantly extended retention times compared to memory devices based on conventional DAE. The loading ratio of DAE0 and DLs was consistently set at 20 wt.% within the F8T2 blend film.

To analyze how different molecular switches affect the memory performance of the OFETs, we prepared bottom‐contact/bottom‐gate OFETs comprising the following four semiconductor layers: F8T2, F8T2/DAE0, F8T2/DL1, and F8T2/DL2. Here DAE0 corresponds to an analogue of DLs used in this study but without photophores and its chemical structure is shown in the inset of **Figure** **4**b. The OFETs with DLs underwent photocrosslinking procedure with a 20% loading ratio of DLs. The loading ratio of the DLs was optimized as shown in Figure S4 (Supporting Information). The prepared semiconductor layers were spin‐coated onto interdigitated Au patterns on an Si/SiO_2_ wafer, with Au serving as the source/drain electrode and an ODTS‐treated Si/SiO_2_ wafer as the gate dielectric/gate electrode. All the samples were post‐annealed at 120 °C for 10 min. As expected, pristine F8T2 OFETs did not show any memory behaviors due to the absence of controllable deep trap states as seen in Figure 4a. Typically, DAE‐embedded polymer semiconductors and their OFETs with high photoprogrammable I_DS_ ON/OFF ratio have not shown memory window behavior because ΔV_th_ as a result of photoisomerization was negligible. However, we found that low crystalline polymer semiconductors such as F8T2 can show large memory window as seen in Figure 4b when an adequate molecular switch (DAE0) is embedded. Notably, large ΔV_th_ shift or memory window over 16 V, along with photoprogrammable I_DS_ ON/OFF ratios surpassing 10^4^ were successfully realized. Similarly, for the F8T2/DL1 OFET, the photoprogrammable I_DS_ ON/OFF ratios over 10 and a memory window of 11 V was observed as shown in Figure 4c. This memory window was further enhanced in the case of F8T2/DL2, exhibiting a memory window of 22 V, as well as photoprogrammable I_DS_ ON/OFF ratios over 10^3^. Both of these values are, so far, the highest values among all DAE‐embedded OFETs (Table S1, Supporting Information).^[^ ^25^, ^26^, ^27^, ^33^, ^48^, ^49^, ^50^, ^51^, ^52^, ^53^, ^54^ ^]^ Figure 4c,d clearly illustrates the significant shift between the initial state and the writing (ON) state, indicating that photocrosslinking induced by DLs affects such a shift of transfer curve. One potential explanation for this observation is that the photophores remaining unreacted undergo further reaction under UV illumination to induce OFF states. Nevertheless, as evident from Figure 4c,d, following the initial writing (ON) / erasing (OFF) step, the transfer curves began to demonstrate stable and repeatable transition between two states.

It is important to note that the application of DAE embedded memory OFETs in digital circuits requires further improvement of the photoprogrammable I_DS_ ON/OFF ratio. To further improve memory performance, one can consider optimizing the chemical structure of DAE and polymer semiconductor or optimizing the thin film morphology or crystallinity to enable deeper trap states. To investigate device‐to‐device variations, we fabricated eight samples from separate batches, and the differences are summarized in Figure S5 (Supporting Information), indicating consistent performance of our device. From the transfer curves, alterations in threshold voltage, drain current of ON state and OFF state for various memory OFET devices were extracted from different batches. Box plots highlight the maximum, minimum, median, and 25‐to‐75 percentile values. Note that the “ON state” corresponds to the open isomeric state of DAE (following visible light irradiation), while the “OFF state” corresponds to the closed isomeric state of DAE (following UV light irradiation).

Both the non‐crosslinked (F8T2/DAE0) and crosslinked (F8T2/DLs) systems exhibited a successful memory window. However, notably, the crosslinked system (F8T2/DLs) displayed a higher observed memory retention time compared to the non‐crosslinked system. As previously mentioned, compared to traditional DAEs, DL1, and DL2 can fix the conjugated backbone of DAE_c in close proximity to the conjugated backbone of the polymer semiconductor through photocrosslinking. Retention characteristics of the ON state and OFF state were measured at a *V*_G_ of –40 V and *V*_D_ of –20 V for the case of F8T2/DAE0 and F8T2/DL2 (Figure 4e,f). Due to non‐crosslinked nature, F8T2/DAE0 OFET rendered very limited memory retention time of less than 10^4^ s, quickly loosing memory characteristics. On the other hand, in the case of F8T2/DL2, the ON and OFF bistable current states were maintained for over 10^6^ s. The extrapolation implies that each state can be distinguished for more than ten years. Notably, the retention behavior of OFF state (DAE_c) was much worse than the case of ON state (DAE_o). This phenomenon occurs due to thermodynamic stability, wherein the open state of DAE exhibits greater stability inherently. In traditional OFET studies, the V_th_ is known to be related to the concentration of deep trap states. Similarly, several nonvolatile memory transistor studies have established that the memory window and memory retention time are determined from the experimental design of the deep trap states.^[^ ^55^ ^]^ However, in previous studies on DAE memory devices, no such memory window characteristics were observed, despite the HOMO level offset between the OSC and DAE_c qualifying as a deep trap.^[^ ^26^ ^]^ Considering these prior studies along with our findings in Figure 4a–d, we propose the following hypothesis: Even if the energy level of DAE_c is in a sufficiently deep trap state compared to the transport energy level of the polymeric semiconductor, an effective energy offset will not result in deep trap states if the physical distance between them is not sufficiently short. In the case of highly crystalline polymer semiconductors, DAEs cannot embed into dense crystalline domain, failing to make DAE_c deep trap states, due to the absence of sufficiently close distance between the conjugated backbone of DAE_c and that of the polymeric semiconductor. In the case of low crystalline polymer semiconductors, DAEs can penetrate loosely packed domain, enabling deep trap states of DAE_c in conjunction with polymer semiconductors. Nevertheless, if chemical bonding between DAEs and polymer semiconductors is not guaranteed, such a deep trap characteristic quickly disappears by phase separation. This assumption is depicted schematically in Figure 4g. To estimate the effective energy offset between the transport levels of F8T2 and the HOMO level of DAE_c, we measured the temperature‐dependent transfer characteristics to extract the activation energy for charge transport *E*_A_, which is related to the energy offset.^[^ ^56^ ^]^ We used the typical Arrhenius equation to estimate *E*_A_:

(1) $$\mu \;=\mu_{0}\;\exp\left(\frac{E_{A}}{kT}\right)$$

where *μ_0_* is the prefactor mobility in limit *T* → *∞*, *k* is the Boltzmann constant, and T is the temperature. We measured the transfer characteristics of F8T2/DL2 after visible (ON state) and UV (OFF state) light irradiation at the corresponding temperatures. To ensure closed isomers for the OFF state, sufficiently long UV illumination was applied before each measurement. As shown in Figure S6 (Supporting Information), the relationship between the extracted charge‐carrier mobility and inverse temperature revealed an Arrhenius‐type *E*_A_ of ≈31.0 and ≈64.7 meV for the ON state and OFF state, respectively. In other words, DAE_c of DL‐2 (corresponding to the OFF state) is a more robust deep trap state than the case of DAE_o (corresponding to the ON state). The measured *E*_A_ value of ≈64.7 meV for DAE_c (OFF state) can be regarded as a sufficiently deep trap state according to previous studies.^[^ ^57^ ^]^

Figure S7 (Supporting Information) illustrates the dynamic switching behavior of F8T2/DL2 OFETs. The reversible current response of the switching behavior was tested using (WRER) cycles. The device remained in the ON state when exposed to visible light for 120 s, while transitioning to the OFF state upon UV light irradiation for 60 s. The ON state–OFF state processes changed the magnitude of I_D_, exhibiting ON/OFF current states over 100 cycles and displaying good reversibility and stability of the memory device. From these results, we can conclude that reliable organic transistor memory devices were successfully fabricated using the F8T2/DL2 thin film, which possesses robust internal stability due to crosslinking.

### 2.6 Photopatterning Performance

As mentioned earlier, we have successfully demonstrated a novel technique for immobilizing blend thin films consisting of the semiconducting polymer F8T2 and the crosslinker DL onto substrates through the precise control of UV light exposure. Conversely, blend thin films that were not subjected to UV light can be easily removed from the substrates by rinsing them with organic solvents. This unique property of the DL crosslinker led us to employ it in a photopatterning process for semiconducting polymers, following the established lithographic approach. To elaborate further, we achieved the photopatterning of the F8T2/DL blend film on a silicon substrate through a two‐step process. First, we spin‐coated the F8T2/DL blend onto the Si substrate at a concentration of 10 wt.% in chloroform (CHCl_3_) at a spinning speed of 2000 rpm. Subsequently, the blended film underwent 10 min of UV light exposure using photomasks to create desired patterns. Following this exposure, we carefully rinsed the film with CHCl_3_ to remove the uncrosslinked areas.

**Figure 5:**
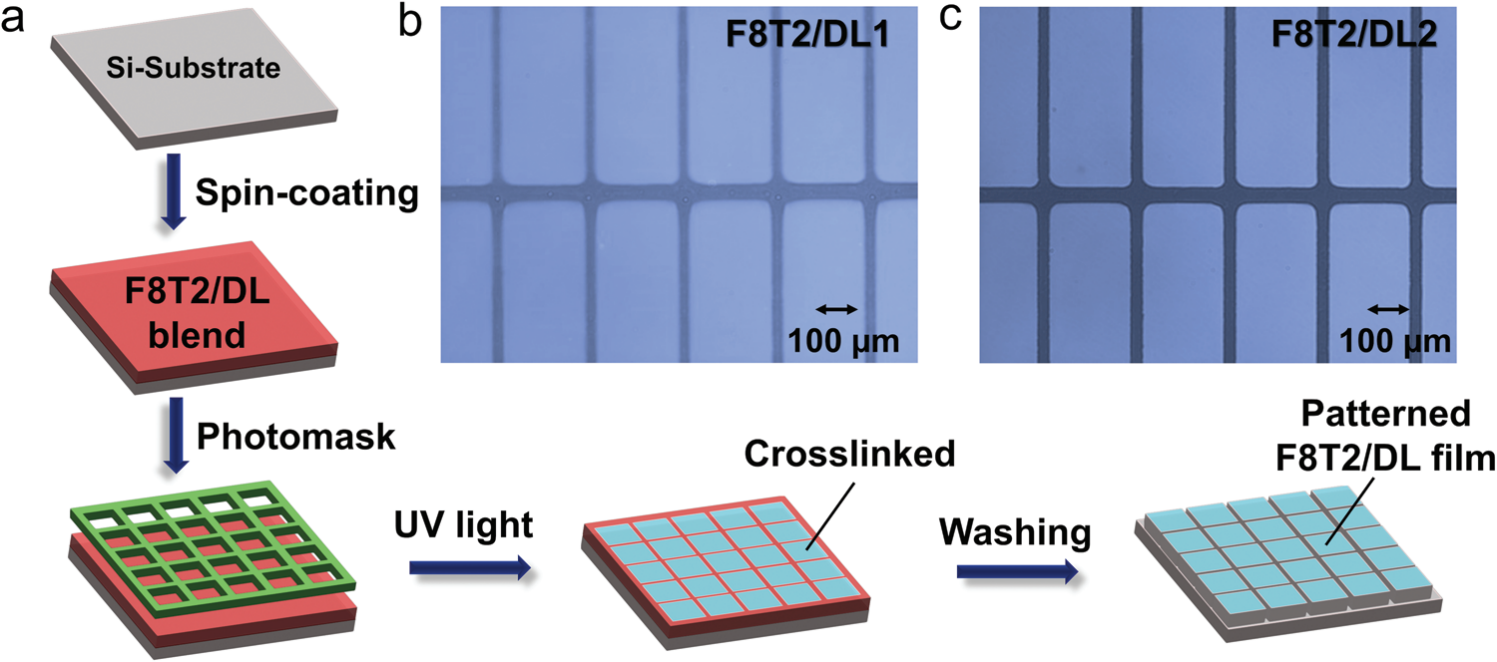
a) Illustration of the photo‐patterning process of F8T2/DL blend film. Optical microscopic image of a striped pattern of F8T2 with a line width of 30 µm b) F8T2/DL1 blend film c) F8T2/DL2 blend film. The weight ratio of the polymer versus DL in the respective blend thin films was 100: 10, and the UV exposure time was 600 s for both of F8T2/DL1 film (254 nm, 0.43 mW cm^−2^) and F8T2/DL2 film (365 nm, 0.47 mW cm^−2^).

By employing a relatively low concentration of just 10 wt.% DL, we were able to create multiple strips of photopatterned F8T2 films with a spacing of 30 µm (**Figure** **5**). This achievement highlights the enormous potential of DL‐mediated photopatterning for generating well‐defined patterns within semiconductor thin films that incorporate molecular switches. These patterned films hold significant promise for applications in diverse fields, including microelectronics and optoelectronics.

The crosslinking efficiency of DL is a crucial factor, allowing for a minimal loading of the material to achieve a crosslinked state in semiconducting polymer films. The photocrosslinking efficiency of DL crosslinker for F8T2 was evaluated indirectly by the film retention, defined as the relative change in the optical absorption at wavelength of 456 nm upon washing with chloroform where the light absorption is most intense for the F8T2 polymer (Figure S8 in Supporting Information). If polymer film is not crosslinked effectively, they would be removed during the rinsing process and thus the film retention would be low. This efficiency is vital when patterning polymer films while retaining their optical and electrical characteristics. Importantly, our photopatterning process does not cause any physical or chemical damage to the F8T2 layer. The UV–vis absorption maxima of F8T2/DL2 at a wavelength of 456 nm remains almost unchanged compared to the original spin‐coated F8T2 film (Figures S2b and S8, Supporting Information). This emphasizes the preservation of the film's electronic properties, rendering it highly suitable for diverse applications. Notably, our DAE‐linker plays a dual role by serving as both a solution‐processable layer and a crosslinker. This unique dual functionality provides us with the capability to create patternable large‐area transistor arrays tailored for future high‐density memory applications.

## 3 Conclusion

This study has addressed the formidable challenge of simultaneously achieving a substantial memory window with a long retention time, a high photoprogrammable ON/OFF ratio, operational stability, and photopatternability in molecular‐switch‐embedded OFETs. For this purpose, an innovative approach has been introduced using photophore‐anchored molecular switches that function as dual‐purpose entities, serving as both molecular switches and photocrosslinkers. The novel design of these molecules coupled with a crosslinking strategy, allows their closed isomeric states to function as morphologically stable and effective deep trap states when combined with polymer semiconductors. This synergy facilitated the attainment of a substantial memory window of 22 V with a long memory retention over 10^6^ s, along with a remarkable photoprogrammable ON/OFF ratio exceeding over 10^3^. This achievement overcomes the limitations observed in previous studies on molecular‐switch‐embedded OFETs. An important observation emerged from the incorporation of a photophore‐anchored molecular switch designated DL2, which contains a diazirine moiety. This compound outperformed its counterpart, DL1, which contains azide, due to more favorable crosslinking conditions associated with DL2. Various characterization techniques were introduced to study the photochemical properties, crosslinking kinetics, film stability, and microstructures of blended films comprising DLs and polymer semiconductors. The inherent versatility of the proposed approach makes it a promising contender for deployment across a spectrum of OFETs that are unbounded by the constraints of the specific chemical architectures of OSCs. This study constitutes a significant advancement in the realm of molecular switch design, holding transformative potential in advanced electronic device fabrication.

## 4 Experimental Section

### Synthesis

DL derivatives were synthesized by lithiation, followed by borylation and subsequent Suzuki cross‐coupling of intermediates as shown in Scheme S1 (Supporting Information). The synthetic details and characterization are given in the Supporting Information.

### Measurement

The ^1^H NMR, ^13^C NMR, and ^19^F NMR spectra were obtained using TMS as the internal standard on Bruker AVANCE‐400 spectrometers with CDCl_3_ as the NMR solvent. A Thermo Scientific Evolution 220 UV–vis spectrophotometer was used to obtain the UV–vis absorption spectra. A Bruker vertex 70v was used to obtain the FTIR spectra. Temperature‐dependent transfer characteristics were measured using a Janis ST‐500‐5CX‐1CXKEL and Keithley 2450 parameter analyzer. Cyclic voltammetry (CV) was employed to investigate the electrochemical behavior of DL1‐o and DL2‐o. The CV experiments were conducted using a potentiostat with a scan rate of 50 mV/s in a three‐electrode cell configuration. In this arrangement, a Pt wire served as the counter electrode, and the reference electrode employed was Ag/AgCl (0.1 m). For sample preparation, a solution of each compound (0.5 mm of DL1‐o and DL2‐o in their open forms) was drop‐cast onto a gold‐disc electrode (1 mm diameter) using CHCl_3_. The deposited solution was allowed to dry at room temperature for 30 min. Subsequently, the electrode was immersed in an acetonitrile solution containing 0.1 m tetrabutylammonium hexafluorophosphate (TBAPF_6_). Figure S9 (Supporting Information) illustrates the CV in the positive potential range, and these results are consistent with prior literature findings.^[^ ^25^ ^]^ The estimated highest occupied molecular orbital (HOMO) levels for the open forms of DL1 and DL2 were determined to be −5.4 and −5.6 eV, respectively. The condition for fabricating thin films was similar to that of OFET devices.

### Device Fabrication

The study utilized n‐doped Si/SiO_2_ (100 nm) wafers as both the substrate and gate for the device, which was designed with a bottom‐contact/bottom‐gate geometry and a W/L ratio of 1580 (with a channel length of 5 µm). Gold electrodes were pre‐patterned onto the substrates. The substrates were cleaned using a sequential process involving detergent (Mucasol), deionized water, acetone, and 2‐propanol, with the use of a sonicating method, and then dried with nitrogen flow. The dried substrates were treated with a UV‐ozone cleaner to eliminate surface contaminants. All substrates were submerged in ODTS (0.3 mL in 60 mL of toluene) for 30 min, rinsed with toluene, dried with nitrogen flow, and annealed at 120 °C for 1 h. Next, a 5 mg mL^−1^ solution of F8T2/DL in chloroform was spin‐coated at 2000 rpm for 1 min. The samples were then subjected to annealing at 120 °C for a period of 10 min. Octadecyl trichloro silane was employed as self‐assembled layer (SAM) treatment on Si/SiO_2_ to ensure optimized charge transport channel between the dielectric and semiconductor layer.^[^ ^60^, ^61^ ^]^

### Device Characterization

To investigate the optoelectrical properties of the OFETs, the study used a semiconductor parameter analyzer (Keysight B1500A) in a nitrogen‐filled glovebox equipped with a UV lamp (312 nm, 0.45 mW cm^−2^) for the OFF state and a visible light (520 nm, 0.15 mW cm^−2^) for the ON state.^[^ ^58^, ^59^ ^]^ The UV irradiation time was set to 60 s, while the visible light irradiation time was set to 120 s. After these irradiation durations, additional exposure at the same wavelength did not result in any additional alteration of the drain current.

## Conflict of Interest

The authors declare no conflict of interest.

## Supporting information

Supporting Information

## Data Availability

The data that support the findings of this study are available from the corresponding author upon reasonable request.
