# Supplementary material for: Photophore‐Anchored Molecular Switch for High‐Performance Nonvolatile Organic Memory Transistor
Source: Adv Sci (Weinh). 2024 Mar 30;11(23):2401482. doi: 10.1002/advs.202401482 (PMC11186055; doi:10.1002/advs.202401482)
Supplement: Supplementary file 1 — Supporting Information [file ADVS-11-2401482-s001.pdf]

ADVANCED SCIENCE
Open Access

Supporting Information

for *Adv. Sci.*, DOI 10.1002/advs.202401482

Photophore-Anchored Molecular Switch for High-Performance Nonvolatile Organic Memory Transistor

*Syed Zahid Hassan, Jieun Kwon, Juhyeok Lee, Hye Ryun Sim, Sanghyeok An, Sangjun Lee and Dae Sung Chung**

Supporting Information

Photophore-Anchored Molecular Switch for High Performance Nonvolatile Organic Memory Transistor

*Syed Zahid Hassan, Jieun Kwon, Juhyeok Lee, Hye Ryun Sim, Sanghyeok An, Sangjun Lee, Dae Sung Chung**

Materials:

All reagents were obtained from commercial suppliers and used without further purification. n-Butyllithium (2.5 M in n-hexane), tributyl borate ($(n\text{-}BuO)_3B$), 4-bromobenzaldehyde, titanium tetrachloride ($TiCl_4$), N-chlorosuccinimide (NCS), $Pd(PPh_3)_4$, acetic acid, N,N'-dicyclohexylcarbodiimide (DCC) (1.0 M in methylene chloride), EtOH, diethyl ether, anhydrous (THF), anhydrous dichloromethane (DCM), anhydrous toluene and $CDCl_3$ were purchased from Sigma Aldrich. 1-Bromo-4-hexylbenzene, 1-bromopropane, glutaryl chloride, aluminum chloride ($AlCl_3$), 4-(dimethylamino)pyridine (DMAP) and sodium borohydride ($NaBH_4$) were purchased from TCI Chemicals. Na_2CO_3, $MgSO_4$, NaOH, Zinc, silica gel, dichloromethane, n-hexane, and ethyl acetate were purchased from Daejung Chemicals Korea. n-Butyllithium is pyrophoric material thus necessary precaution should be taken while handling. 4-methyl-2-propylthiophene **(1)**, 4-azido-2,3,5,6-tetrafluorobenzoic acid, and 4-[3-(trifluoromethyl)-3H-diazirin-3-yl]benzoic acid were synthesized in our laboratory following the earlier report.[1-3]

Scheme S1. Synthetic scheme for DL1 and DL2.

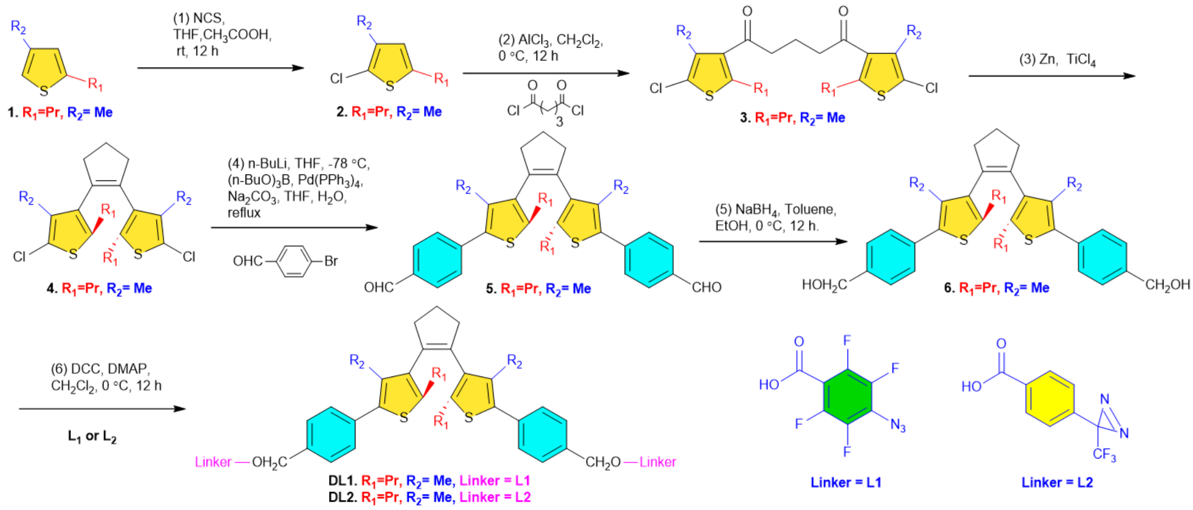

2-chloro-3-methyl-5-propylthiophene (2)

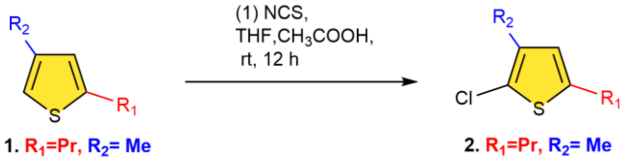

N-chlorosuccinimide (5.24 g, 39.2 mmol) was added to a solution of 4-methyl-2-propylthiophene **(1)** (5 g, 35.6 mmol) in the stirred solution of dry THF (30 mL) and acetic acid (30 mL) while continuously purging N_2 gas at 0 °C. After 1 h, the reaction mixture was heated at 60 °C for 4 h. The cooled reaction mixture was poured into an aqueous solution of NaOH, extracted with diethyl ether, washed with water, dried over $MgSO_4$, and concentrated under vacuum. Column chromatography (silica gel, n-hexane) provided 2-chloro-3-methyl-5-propylthiophene **(2)** (4.1 g, 23.4 mmol, colorless liquid) in 65 % yield.

^{1}H NMR (TMS, 400 MHz, $CDCl_3$) δ = 0.99 (t, 3H), 1.68 (m, 2H), 2.16 (s, 3H), 2.69 (t, 2H), 6.49 (s, 1H).

^{13}C NMR (100 MHz, $CDCl_3$) δ = 13.51, 13.60, 24.66, 32.32, 125.89, 128.64, 133.64, 141.92.

1,5-Bis(2-chloro-3-methyl-5-propylthien-3-yl)pentane-1,5-dione (3)

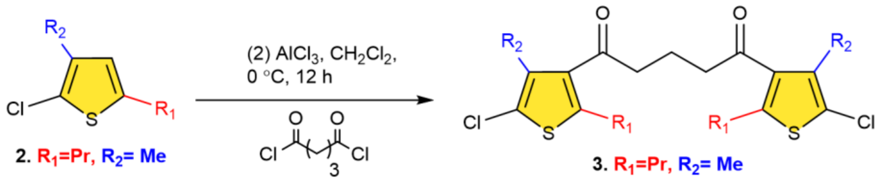

Glutaryl chloride (1.32 mL, 10.3 mmol), and 2-chloro-3-methyl-5-propylthiophene **(2)** (4.0 g, 22.9 mmol) were added to CH_2Cl_2 (100 mL) under stirring. The temperature was reduced to 0 °C, followed by the slow addition of $AlCl_3$ (4.58 g, 34.3 mmol). The reaction mixture was stirred for 2 h at room temperature, followed by neutralization of excess $AlCl_3$ by the addition of cold H_2O (50 mL). The crude product was extracted with CH_2Cl_2, dried over $MgSO_4$, and the solvent was evaporated under vacuum. Column chromatography (silica gel, n-hexane: EtOAc = 10:1) afforded pure 1,5-bis(2-chloro-3-methyl-5-propylthien-3-yl)pentane-1,5-dione **(3)** (2.53 g, 5.6 mmol, colorless liquid) in 54 % yield.

^{1}H NMR (TMS, 400 MHz, $CDCl_3$) δ = 0.97 (t, 6H), 1.64 (s, 4H), 2.07 (m, 2H), 2.18 (s, 6H), 2.80 (m, 8H).

^{13}C NMR (100 MHz, $CDCl_3$) δ = 13.79, 13.84, 18.72, 25.10, 31.47, 42.59, 122.61, 132.40, 138.31, 145.46, 200.33.

1,2-Bis(2-chloro-3-methyl-5-propylthien-3-yl)cyclopentene (4)

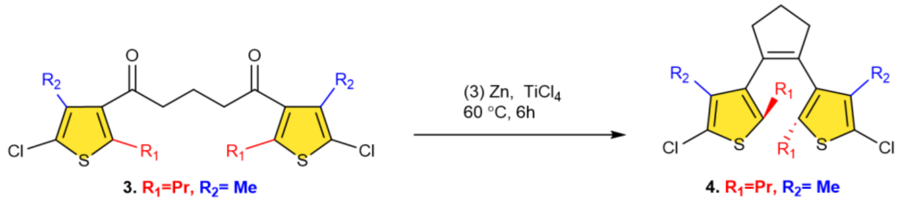

$TiCl_4$ (3.41 g, 17.9 mmol) and powdered zinc (1.76 g, 26.9 mmol) were added under nitrogen in dry THF (100 mL) at 0 °C under stirring followed by reflux for 2 h. Then, the temperature was reduced to 0 °C and 1,5-bis(2-chloro-3-methyl-5-propylthien-3-yl)pentane-1,5-dione **(3)** (2.0 g, 4.4 mmol) was added to the solution. After stirring for 6 h at 60 °C, the mixture was poured into saturated aqueous K_2CO_3. After extraction with diethyl ether (3 × 50 mL), the combined diethyl ether layers were washed with water, dried over $MgSO_4$, and filtered, and the solvent was evaporated under vacuum to yield a brown oil. Chromatography on silica gel (n-hexane) afforded the product as a yellow liquid (1.15 g, 2.7 mmol, 61%).

^{1}H NMR (TMS, 400 MHz, $CDCl_3$) δ = 0.84 (m, 6H), 1.44 (b, 4H), 2.06 (b, 2H), 2.13 (s, 6H), 2.69 (b, 8H).

^{13}C NMR (100 MHz, $CDCl_3$) δ = 13.83, 23.85, 30.79, 37.75, 121.36, 132.73, 133.94, 137.18, 137.75.

1,2-Bis(4-methyl-2-propyl-5-(4-formylphenyl)-3-thienyl)cyclopentene (5)

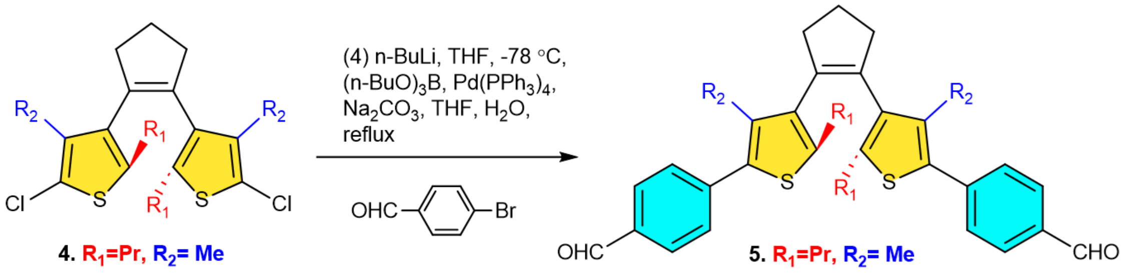

n-Butyllithium (2.5 M in n-hexane, 2.03 mL, 5.0 mmol) was added dropwise to a solution of 1,2-bis(2-chloro-3-methyl-5-propylthien-3-yl)cyclopentene **(4)** (1.0 g, 2.42 mmol) in dry THF (30 mL) while continuously purging N_2 gas at –78 °C. After 1 h, tributyl borate (1.42 mL, 5.3 mmol) at –78 °C was added dropwise. After 1 h, the reaction mixture was brought back to room temperature. The obtained reaction mixture was used in the next step of the Suzuki–Miyaura coupling. 4-Bromobenzaldehyde (0.94 g, 5.0 mmol), Na_2CO_3 (0.51 g, 4.8 mmol), water (4 mL)

and $Pd(PPh_3)_4$ (55 mg, 0.048 mmol) were added to the reaction mixture obtained earlier, and the mixture was heated under reflux for 24 h. The reaction mixture was extracted with dichloromethane, washed with water, dried over $MgSO_4$, and concentrated in vacuo. Column chromatography (silica gel, n-hexane/EtOAC = 5/1) gave 1,2-bis-(4-methyl-2-propyl-5-(4-formylphenyl)-3-thienyl)cyclopentene **(5)** (0.63 g, 1.1 mmol, light brown solid) in 47 % yield.

^{1}H NMR (TMS, 400 MHz, $CDCl_3$) δ = 0.80 (t, 6H), 1.44 (br, 4H), 2.19 (br, 2H), 2.33 (s, 6H), 2.60 (br, 8H), 7.60 (d, 4H, 7.92 (d, 4H), 10.04 (s, 2H).

^{13}C NMR (100 MHz, $CDCl_3$) δ = 14.08, 15.75, 24.02, 24.60, 31.02, 37.99, 129.10, 129.90, 133.67, 134.43, 136.75, 137.75, 141.56, 191.68.

<u>1,2-Bis-(4-methyl-2-propyl-5-(4-methanoylphenyl)-3-thienyl)cyclopentene (6)</u>

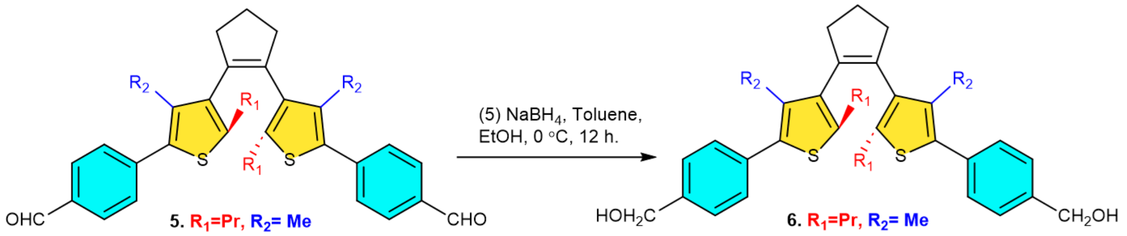

1,2-bis-(4-methyl-2-propyl-5-(4-formylphenyl)-3-thienyl)cyclopentene **(5)** (0.60 g, 1.0 mmol) was dissolved in dry toluene (20 mL) and diluted with ethanol (20 mL) under stirring. Subsequently, sodium borohydride (123 mg, 3.2 mmol) was slowly added and stirring was continued for 2 h at room temperature. The reaction mixture was neutralized by adding water slowly, followed by acidification with HCl. The reaction mixture was extracted with dichloromethane, dried over $MgSO_4$, and concentrated in vacuo. Column chromatography (silica gel, n-hexane/EtOAC = 3/1) gave 1,2-bis-(4-methyl-2-propyl-5-(4-methanoylphenyl)-3-thienyl)cyclopentene **(6)** (0.52 g, 0.9 mmol, 86 %).

^{1}H NMR (TMS, 400 MHz, $CDCl_3$) δ = 0.79 (t, 6H), 1.44 (br, 4H), 2.02 (d, 2H), 2.28 (s, 6H), 2.62 (br, 8H), 4.74 (s, 4H), 7.41 (m, 8H).

^{13}**C NMR** (100 MHz, $CDCl_3$) δ = 14.19, 15.39, 24.07, 24.66, 30.93, 37.95, 65.13, 125.31, 127.10, 128.24, 129.22, 131.97, 134.33, 134.80, 136.13, 137.59, 139.28, 139.83.

1,2-Bis-(4-methyl-2-propyl-5-(methylphenyl-4-azido-2,3,5,6-tetrafluorobenzoate)-3-thienyl)cyclopentene (DL1)

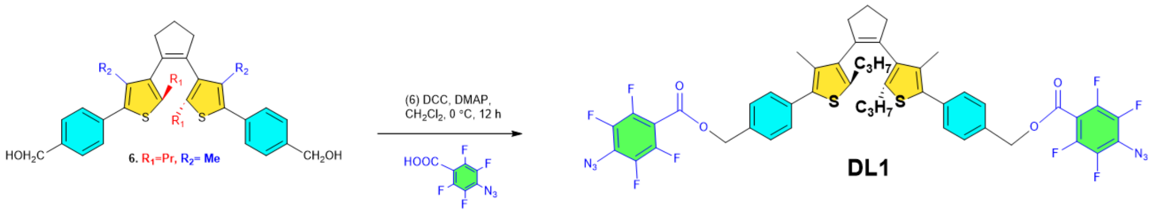

A mixture of 4-azido-2,3,5,6-tetrafluorobenzoic acid (0.25 g, 1.0 mmol) and 1,2-bis-(4-methyl-2-propyl-5-(4-methanoylphenyl)-3-thienyl)cyclopentene **(6)** (0.20 g, 0.36 mmol) were dissolved in anhydrous dichloromethane (25 mL) under stirring at room temperature followed by addition of DMAP (9 mg, 71 μmol). After 30 min, the temperature was lowered to 0 °C, and DCC (1 M in dichloromethane) (0.79 mL, 0.79 mmol) was added under a N_2 atmosphere. After 12 h, the reaction mixture was neutralized with water, extracted with dichloromethane, and the solvent was removed by distillation at reduced pressure. The resulting crude product was purified by silica gel column chromatography using an eluent of ethyl acetate/n-hexane (1/5), which yielded the **DL1** as a white solid (0.16 g, 0.16 mmol, 44 %).

^{1}**H NMR** (TMS, 400 MHz, $CDCl_3$) δ = 0.79 (t, 6H), 1.42 (b, 4H), 2.02 (m, 2H), 2.28 (s, 6H), 2.43 (b, 2H), 2.62 (b, 2H), 2.79 (b, 2H), 2.98 (b, 2H), 5.44 (s, 4H), 7.46 (m, 8H).

^{13}**C NMR** (100 MHz, $CDCl_3$) δ = 14.14, 15.40, 24.04, 24.62, 30.88, 37.94, 68.02, 107.58, 128.42, 129.25, 132.26, 132.94, 133.96, 135.84, 136.22, 137.60, 140.14, 152.04, 159.23.

^{19}**F NMR** (100 MHz, $CDCl_3$) δ = −138.30, −150.91.

<u>1,2-bis-(4-methyl-2-propyl-5-(methylphenyl-4-[3-(Trifluoromethyl)-3H-diazirin-3-yl]benzoate)-3-thienyl)cyclopentene (DL2)</u>

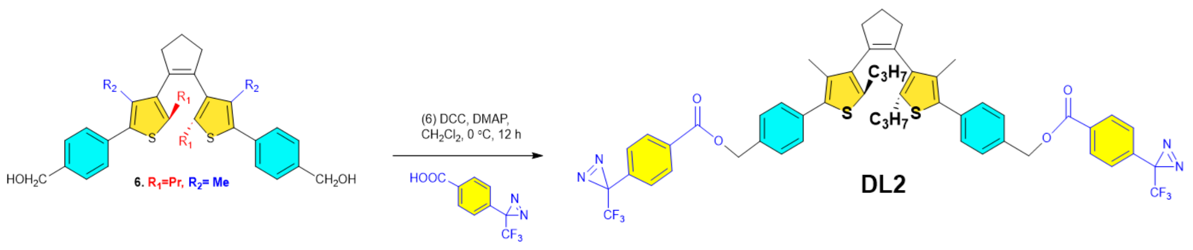

A mixture of 4-[3-(trifluoromethyl)-3 H -diazirin-3-yl]benzoic Acid (0.24 g, 1.0 mmol) and 1,2-bis-(4-methyl-2-propyl-5-(4-methanoylphenyl)-3-thienyl)cyclopentene **(6)** (0.20 g, 0.36 mmol) were dissolved in anhydrous dichloromethane (25 mL) under stirring at room temperature followed by addition of DMAP (9 mg, 71 μmol). After 30 min, the temperature was lowered to 0 °C, and DCC (1 M in dichloromethane) (0.79 mL, 0.79 mmol) was added under a N_2 atmosphere. After 12 h, the reaction mixture was neutralized with water, extracted with dichloromethane, and the solvent was removed by distillation at reduced pressure. The resulting crude product was purified by silica gel column chromatography using an eluent of ethyl acetate/n-hexane (1/5), which yielded the **DL2** as a white solid (0.18 g, 0.18 mmol, 51 %).

^{1}H NMR (TMS, 400 MHz, $CDCl_3$) δ = 0.79 (t, 6H), 1.30 (b, 4H), 2.01 (b, 2H), 2.27 (6H, s), 2.42 (b, 2H), 2.61 (b, 2H), 2.79 (b, 2H), 2.96 (b, 2H), 5.41 (s, 4H), 7.27 (d, 4H), 7.45 (m, 8H), 8.14 (d, 4H).

^{13}C NMR (100 MHz, $CDCl_3$) δ = 14.13, 15.35, 24.04, 24.59, 30.92, 37.96, 66.85, 126.39, 128.32, 129.23, 130.06, 131.23, 132.19, 133.89, 134.03, 135.61, 136.22, 137.60, 165.38.

^{19}F NMR (100 MHz, $CDCl_3$) δ = −64.95.

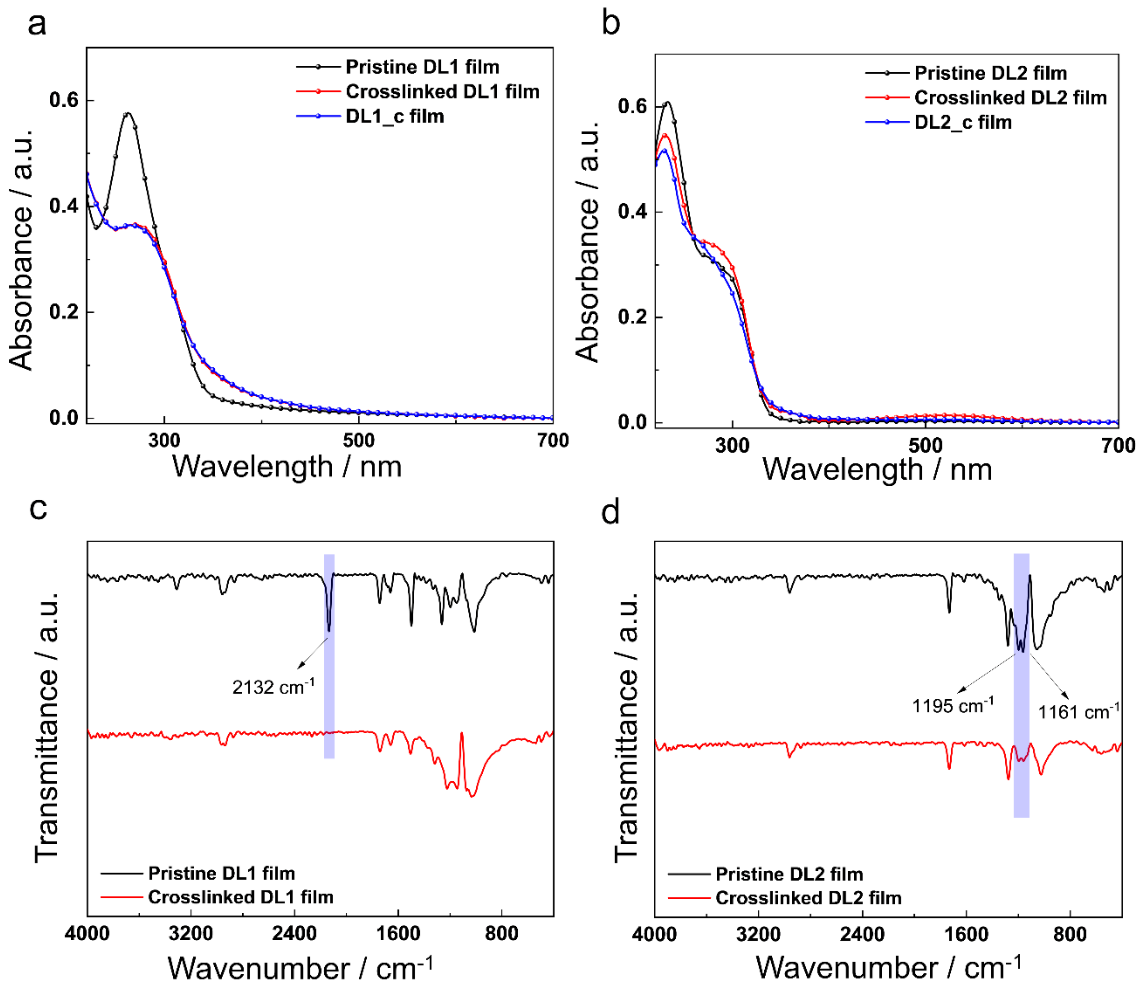

Figure S1. UV–vis absorption spectra of the (a) DL1 film, (b) DL2 film pre- and post-photocrosslinking, and subsequent irradiation with 312 nm UV light to induce closed form of DL1 and DL2. FTIR spectra illustrates the absence of azide peak in (c) pristine DL1 film and diazirine peak in (d) pristine DL2 after photocrosslinking.

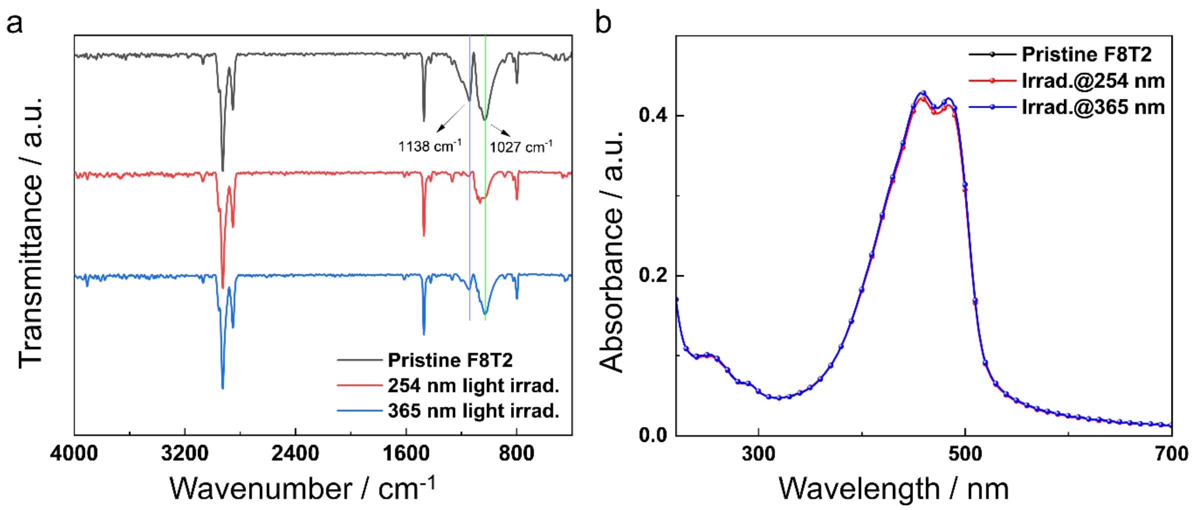

Figure S2. (a) FTIR spectra of the F8T2 film before and after UV light irradiation (254 nm and 365 nm), (b) UV–vis absorption spectra of the F8T2 film before and after UV light irradiation (254 nm and 365 nm).

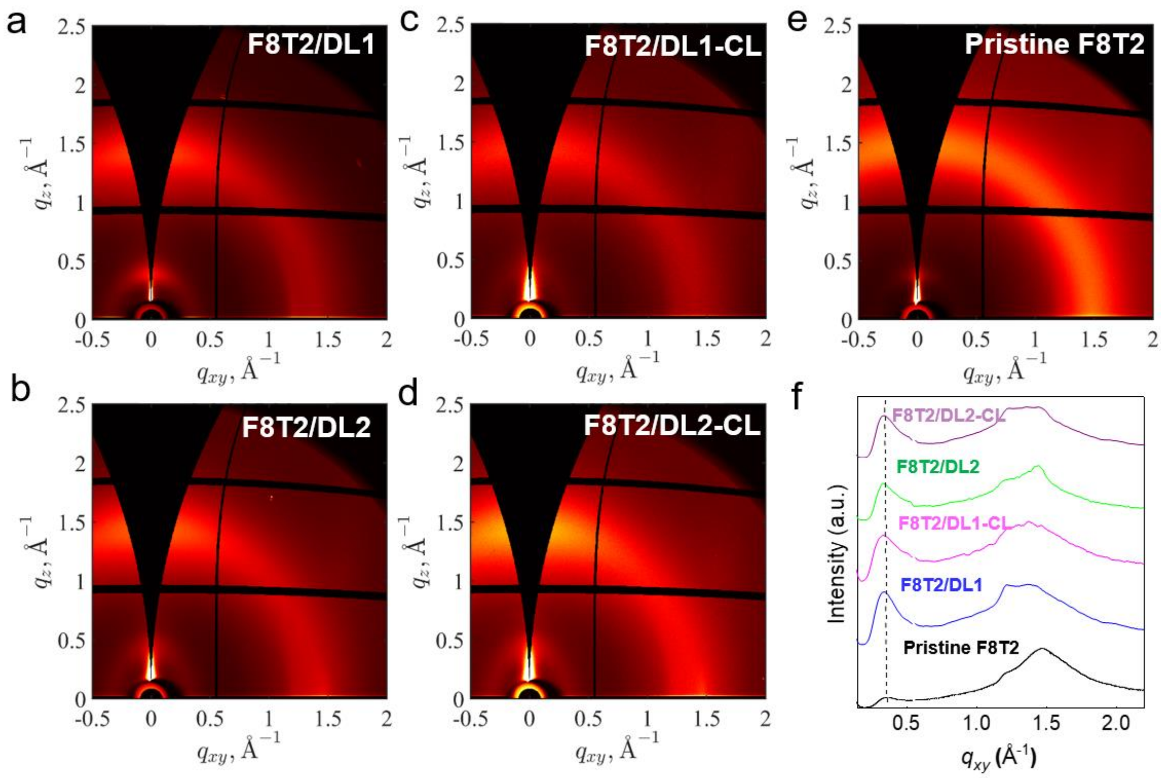

Figure S3. Two-dimensional GIXD images of (a) F8T2/DL1 blend film, (b) F8T2/DL2 blend film, (c) crosslinked F8T2/DL1 blend film, (d) crosslinked F8T2/DL2 blend film, (e) pristine F8T2 films, and (f) corresponding line cut profiles along with in-plane direction. All GIXD images have been corrected for the "missing wedge" of data along the in-plane direction.

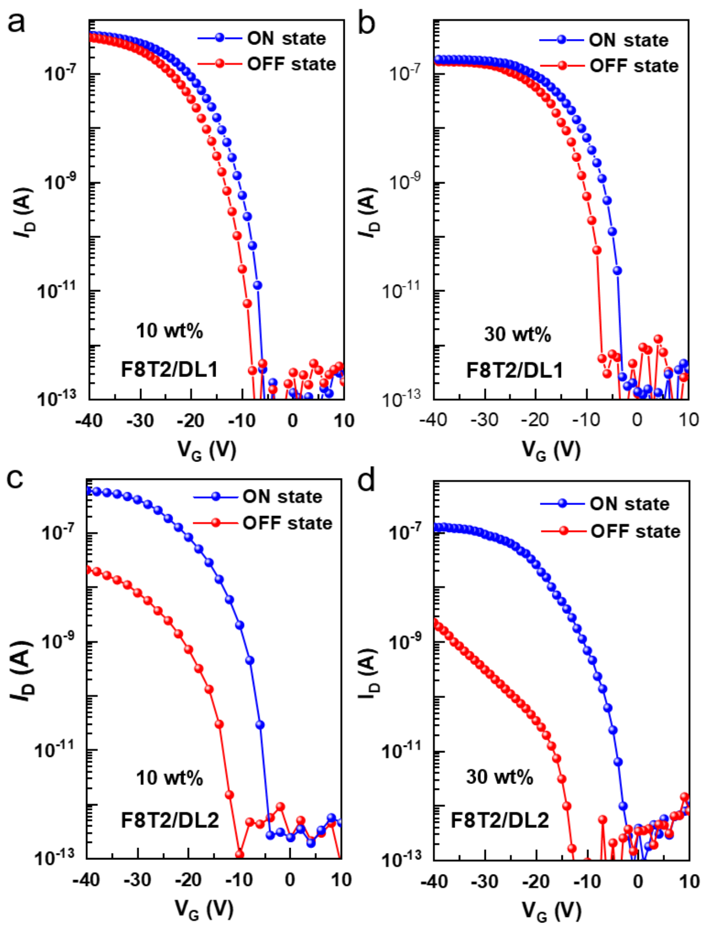

Figure S4. Transfer curve of F8T2/DL blend FETs before and after UV exposure with different blend ratio of 10wt% and 30wt%.

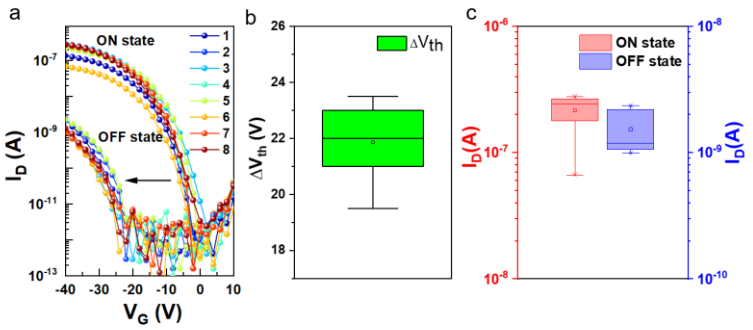

Figure S5. (a) Illustration of the transfer curve for eight distinct batch devices, (b) Box plot presenting the variation in threshold voltage across the eight different batch FET devices, and (c) Box plot summarizing the changes in ON state and OFF state drain current values for the eight different batch FET devices. All measurements were conducted under identical conditions for each of the eight individual devices. The box represents the 25-to-75 percentile range, with whiskers indicating the minimum and maximum values. The median value is denoted by the bar inside the box, and the mean value is marked by the ▫ sign.

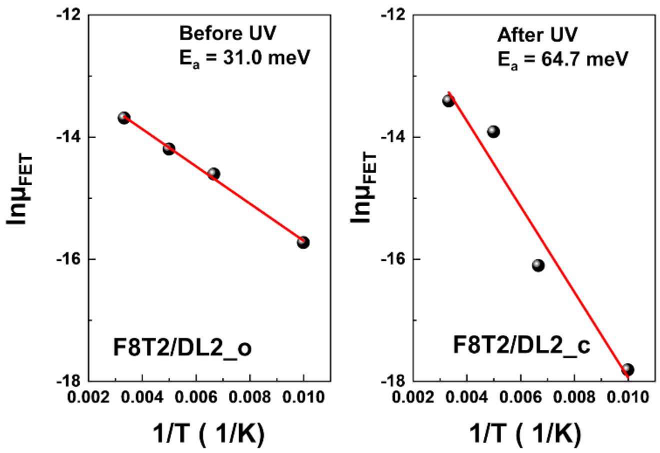

Figure S6. The relationship between the extracted charge carrier mobility of F8T2/DL2 based FET and inverse temperature to obtain Arrhenius type activation energy offset. The obtained activation energy (E_a) value in the closed form of DL2 indicates the presence of a deep trap relative to the open form of DL2.

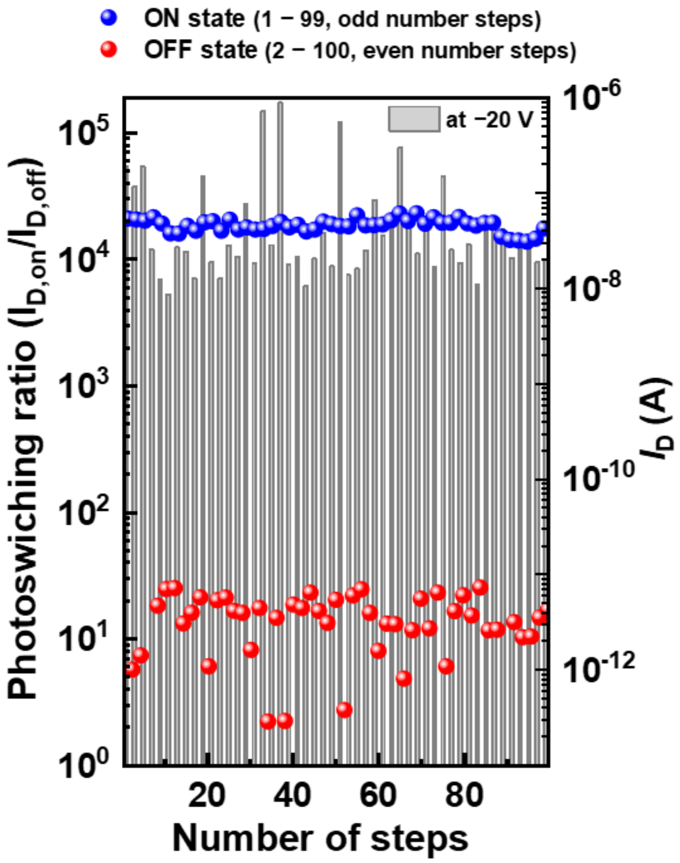

Figure S7. Reversible WRER cycles of the nonvolatile FET memory device for upto 100 photocycles. The photoprogrammed reversible switching ratio and I_{DS} values were extracted from the transfer curves at $V_G = -20$ V and $V_D = -20$ V. The UV irradiation time was fixed at 60 s, and the visible light irradiation time was fixed at 120 s.

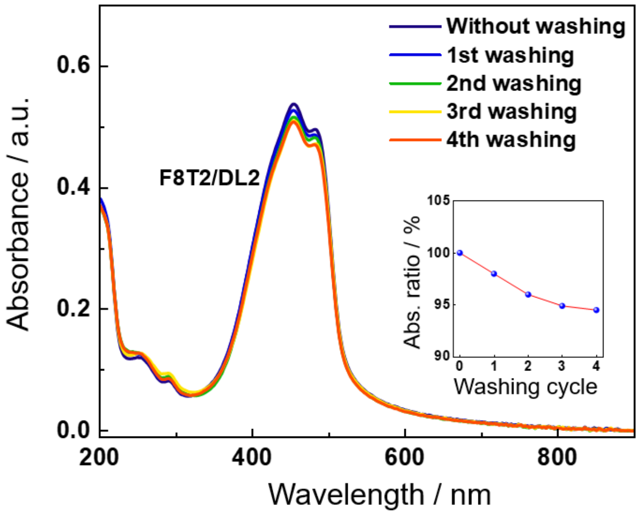

Figure S8. UV-vis absorption spectra of the F8T2/DL2 blend film before and after crosslinking. The inset shows a plot of the absorption ratio vs. number of washing cycles.

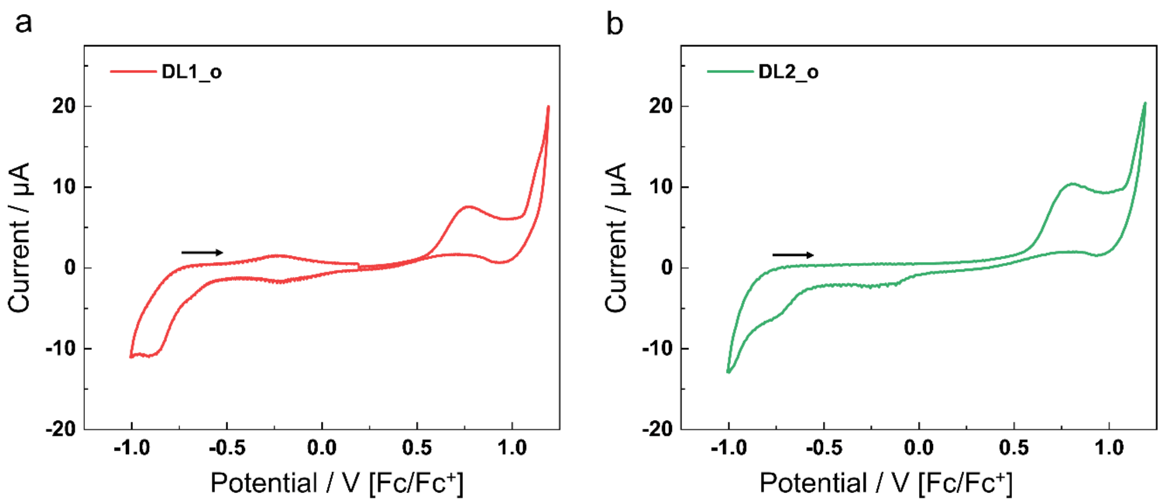

Figure S9. Cyclic voltammetry of (a) DL1_o and (b) DL2_o in acetonitrile / 0.1 M Bu_4NPF_6.

Table S1. Summary of photo-achievable memory window of DAE-embedded OFETs.

System	ΔVth	Maximum photoprogrammable I_{DS} ON/OFF ratio	Maximum number of repeating steps	Retention time (days)	References
P3HT/DAE-Me	15 V	10	70	500	4
F8T2/DAE-Me	< 20 V [a)]	10^3 [b)]	4	N/A	5
DPP-TT/DAE-316	0 V [a)]	10^3 [b)]	100	N/A	2
PCbD-IDTTP/DAE-316	0 V [a)]	10^3 [b)]	150	N/A	6
F8T2/DL2	22 V	10^3	100	>3000	This work

[a)] Estimated values from the transfer curve; [b)] Estimated values from normalized drain–source current in repeating step.

^{1}H NMR of 2

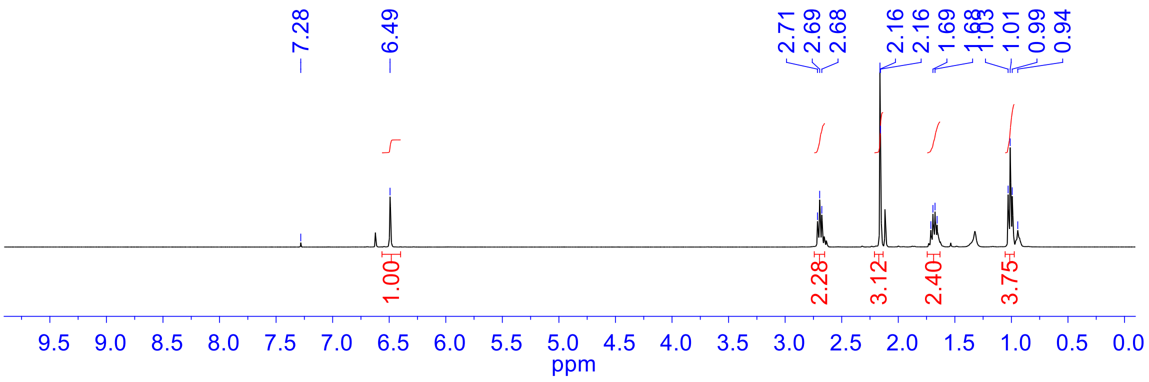

^{13}C NMR of 2

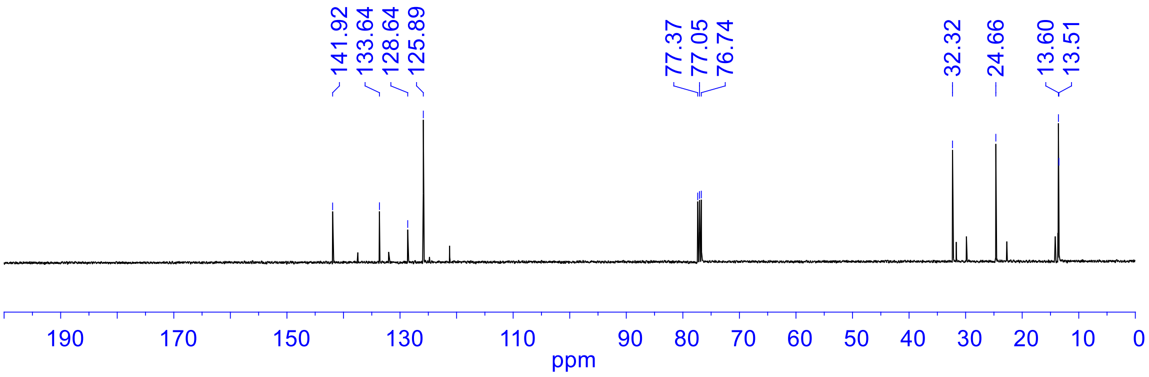

^{1}H NMR of 3

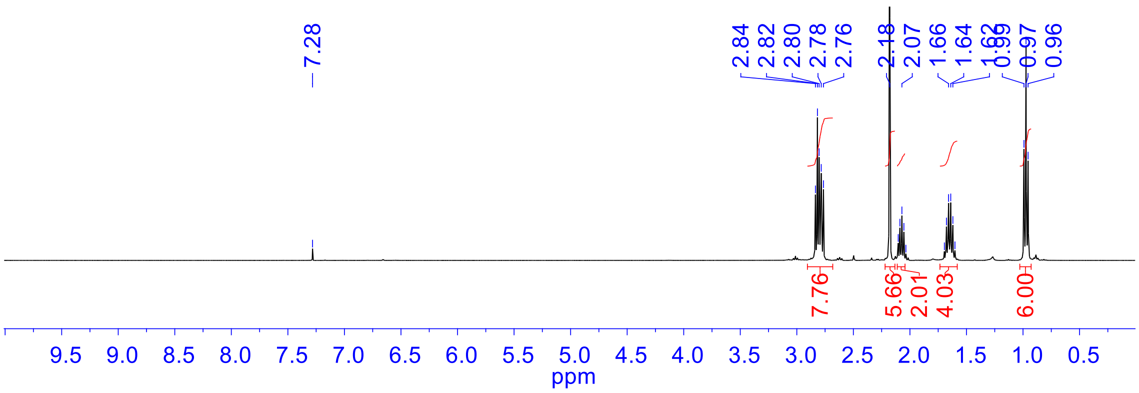

^{13}C NMR of 3

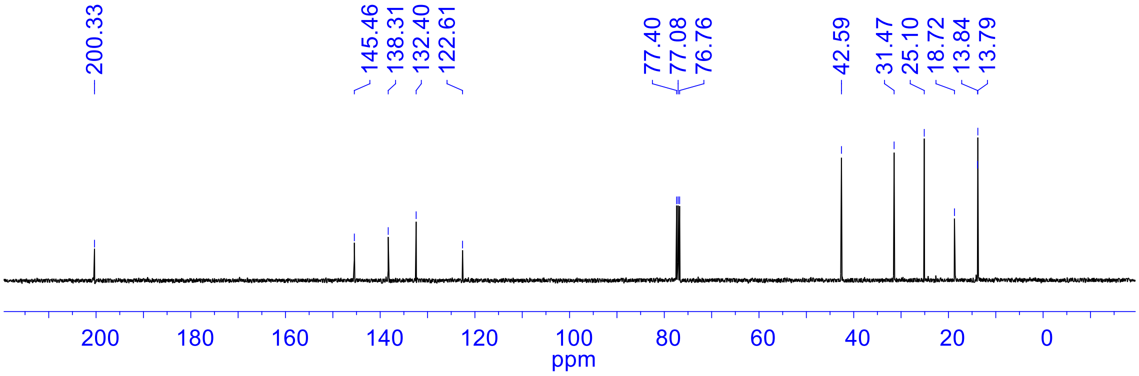

^{1}H NMR of 4

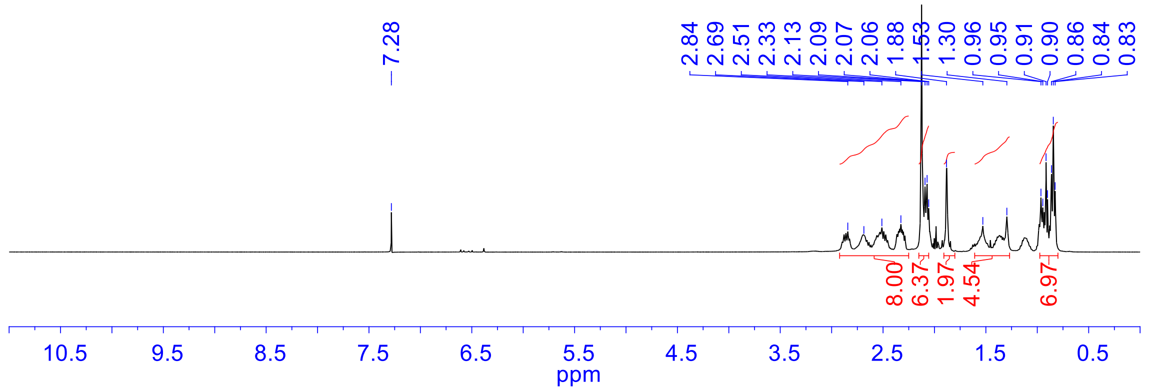

^{13}C NMR of 4

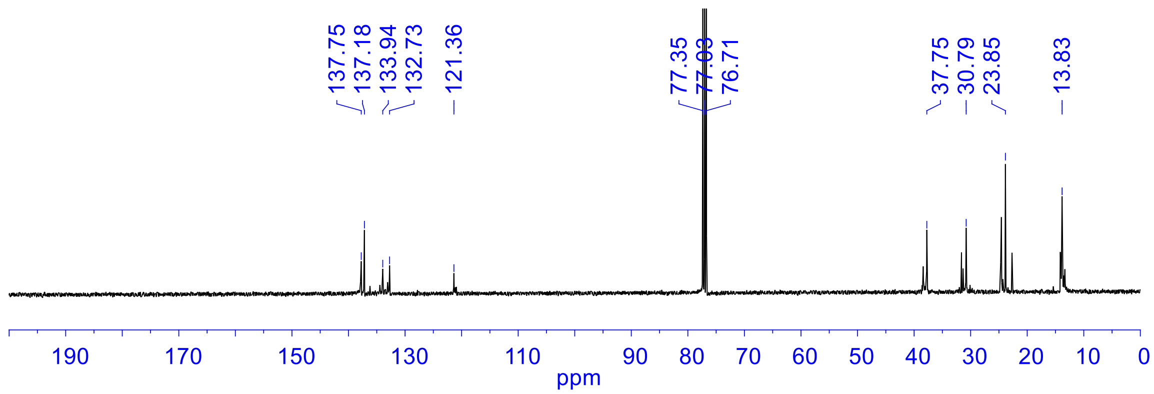

^{1}H NMR of 5

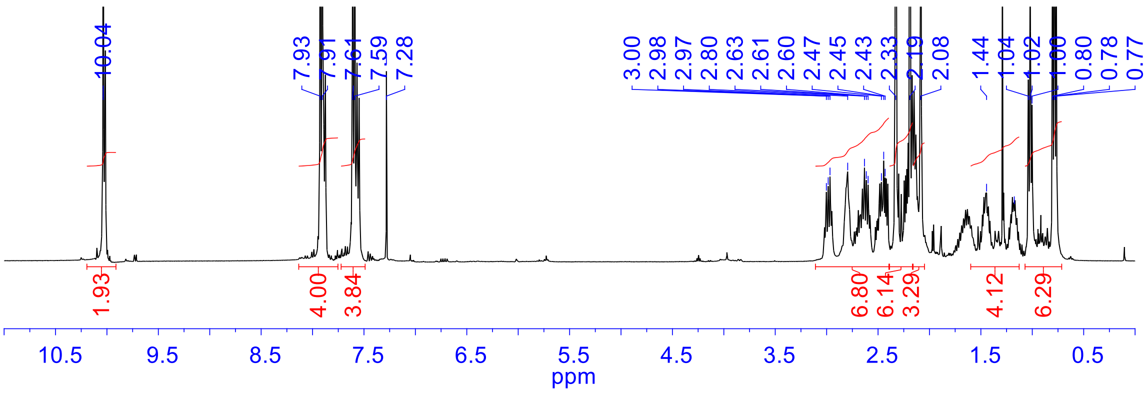

^{13}C NMR of 5

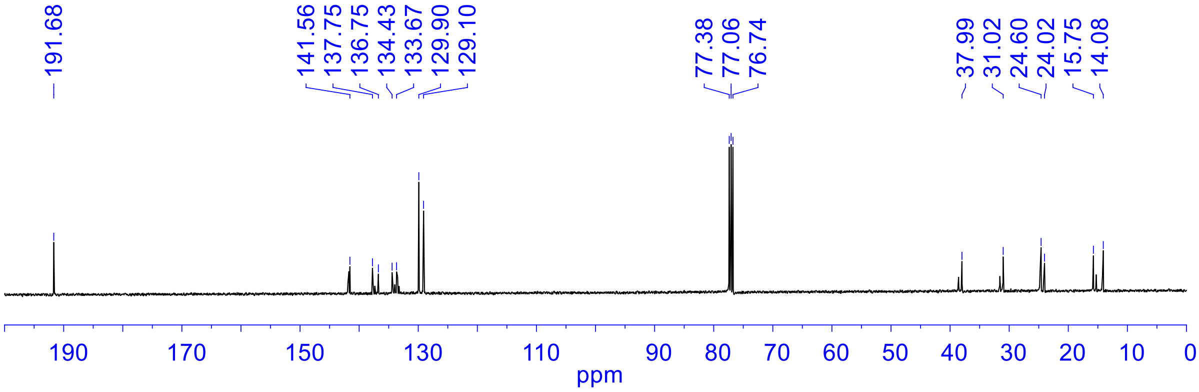

^{1}H NMR of 6

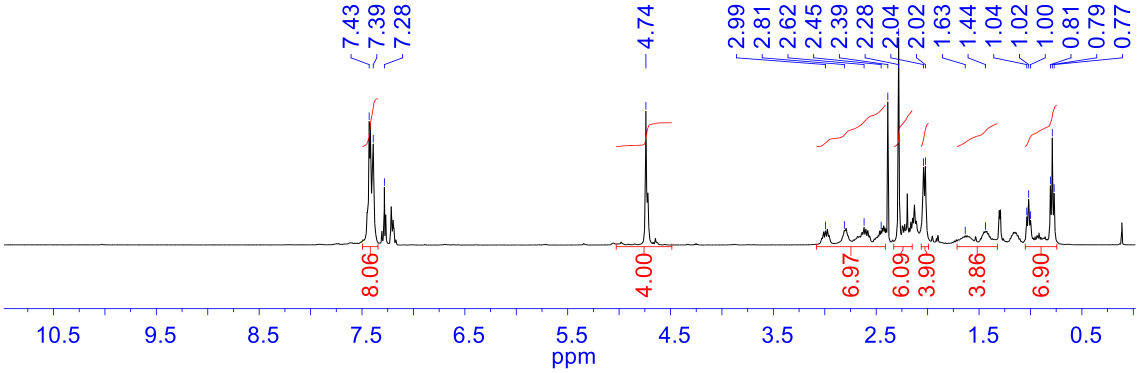

^{13}C NMR of 6

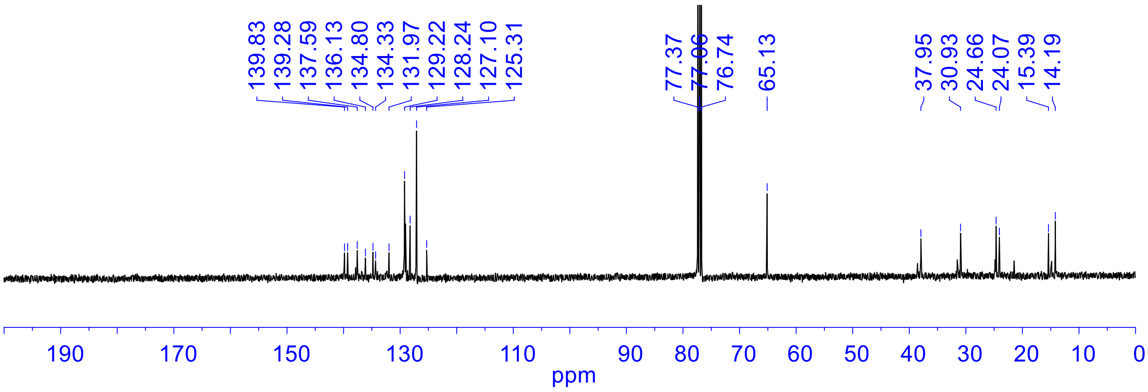

^{1}H NMR of DL1

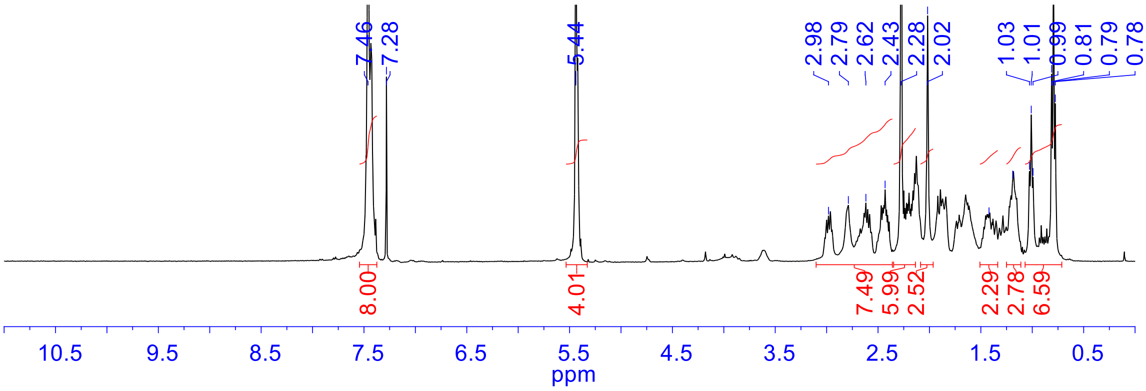

^{13}C NMR of DL1

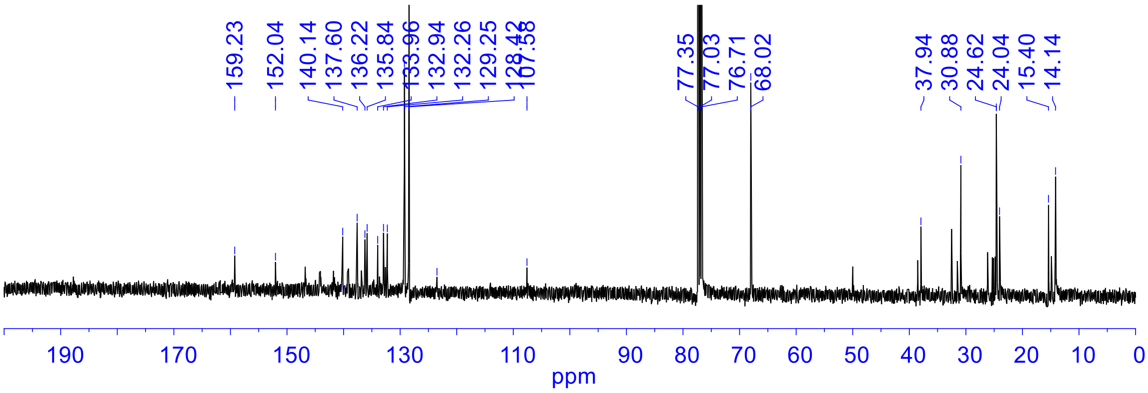

^{19}F NMR of DL1

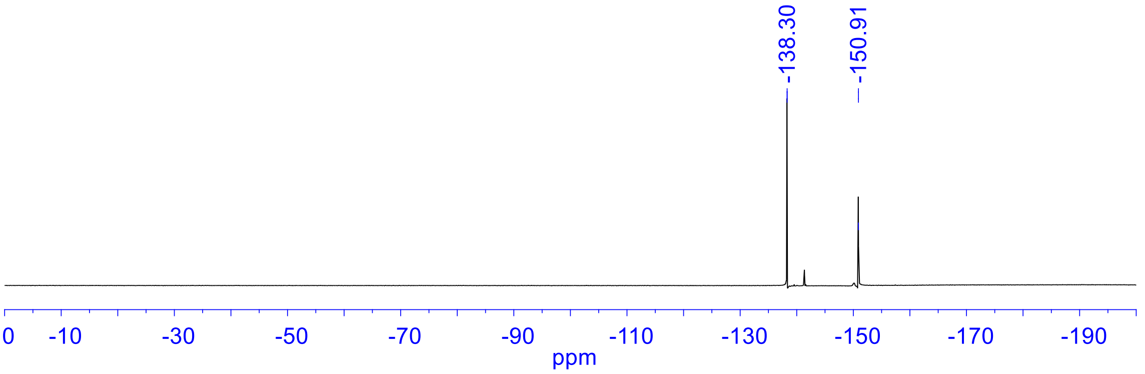

^{1}H NMR of DL2

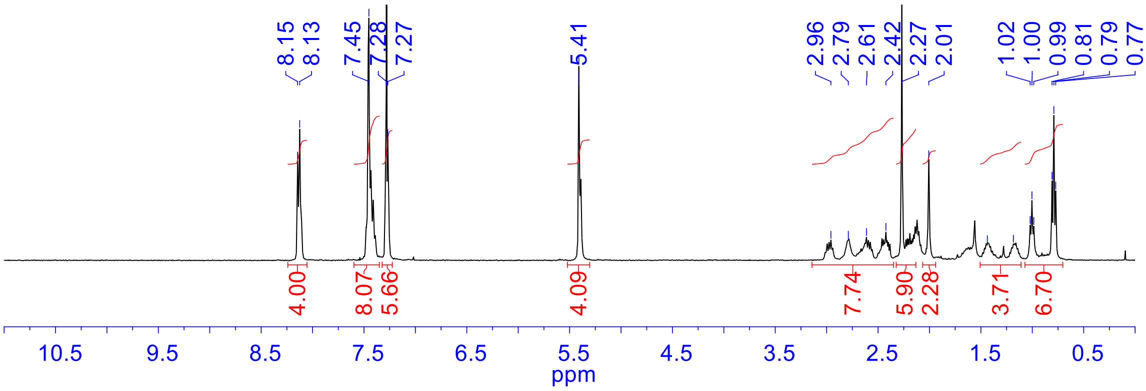

^{13}C NMR of DL2

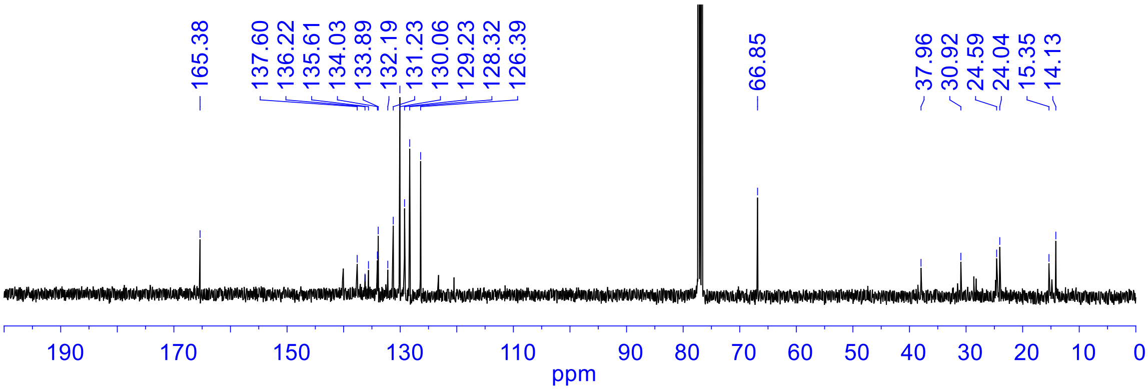

^{19}F NMR of DL2

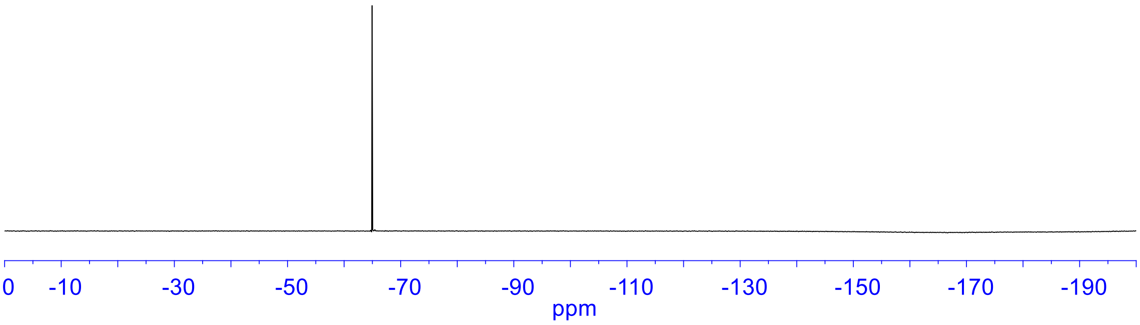

References:

[1] J. Kim, C. W. Joo, S. Z. Hassan, S. H. Yu, M. Kang, J.-E. Pi, S.-Y. Kang, Y.-S. Park, D. S. Chung, *Mater. Horiz.* **2021**, *8*, 3141.

[2] S. Z. Hassan, J. Song, S. H. Yu, D. S. Chung, *Chem. Mater.* **2021**, *33*, 7546.

[3] K. Sakurai, T. Yasui, S. Mizuno, *Asian J. Org. Chem.* **2015**, *4*, 724.

[4] T. Leydecker, M. Herder, E. Pavlica, G. Bratina, S. Hecht, E. Orgiu, P. Samorì, *Nat. Nanotechnol.* **2016**, *11*, 769.

[5] L. Hou, T. Leydecker, X. Zhang, W. Rekab, M. Herder, C. Cendra, S. Hecht, I. McCulloch, A. Salleo, E. Orgiu, P. Samorì, *J. Am. Chem. Soc.* **2020**, *142*, 11050.

[6] S. H. Yu, S. Z. Hassan, S. Lee, B. Lim and D. S. Chung, *J. Mater. Chem. C*, **2023**,*11*, 1560.
